# Aldehyde dehydrogenase 3A1 deficiency leads to mitochondrial dysfunction and impacts salivary gland stem cell phenotype

**DOI:** 10.1093/pnasnexus/pgac056

**Published:** 2022-06-09

**Authors:** Vignesh Viswanathan, Hongbin Cao, Julie Saiki, Dadi Jiang, Aaron Mattingly, Dhanya Nambiar, Joshua Bloomstein, Yang Li, Sizun Jiang, Manish Chamoli, Davud Sirjani, Michael Kaplan, F Christopher Holsinger, Rachel Liang, Rie Von Eyben, Haowen Jiang, Li Guan, Edward Lagory, Zhiping Feng, Garry Nolan, Jiangbin Ye, Nicholas Denko, Sarah Knox, Daria-Mochly Rosen, Quynh-Thu Le

**Affiliations:** Department of Radiation Oncology, Stanford School of Medicine, Stanford, CA 94305, USA; Department of Radiation Oncology, Stanford School of Medicine, Stanford, CA 94305, USA; Department of Radiation Oncology, Stanford School of Medicine, Stanford, CA 94305, USA; Department of Radiation Oncology, The University of Texas MD Anderson Cancer Center, Houston, TX 77030, USA; Department of Cell and Tissue Biology, University of California San Francisco, San Francisco, CA 94143, USA; Department of Radiation Oncology, Stanford School of Medicine, Stanford, CA 94305, USA; Department of Radiation Oncology, Stanford School of Medicine, Stanford, CA 94305, USA; Department of Radiation Oncology, Stanford School of Medicine, Stanford, CA 94305, USA; Department of Microbiology and Immunology, Stanford University School of Medicine, Stanford, CA 94305, USA; Buck Institute for Research on Aging, 8001 Redwood Blvd., Novato, CA 94945, USA; Department of Otolaryngology–Head and Neck Surgery, Stanford University School of Medicine, Stanford, CA 94305, USA; Department of Otolaryngology–Head and Neck Surgery, Stanford University School of Medicine, Stanford, CA 94305, USA; Department of Otolaryngology–Head and Neck Surgery, Stanford University School of Medicine, Stanford, CA 94305, USA; Department of Radiation Oncology, Stanford School of Medicine, Stanford, CA 94305, USA; Department of Radiation Oncology, Stanford School of Medicine, Stanford, CA 94305, USA; Department of Radiation Oncology, Stanford School of Medicine, Stanford, CA 94305, USA; Department of Radiation Oncology, Stanford School of Medicine, Stanford, CA 94305, USA; Department of Radiation Oncology, Stanford School of Medicine, Stanford, CA 94305, USA; Department of Chemical and Systems Biology, Stanford University School of Medicine, Stanford, CA 94305, USA; Department of Microbiology and Immunology, Stanford University School of Medicine, Stanford, CA 94305, USA; Department of Radiation Oncology, Stanford School of Medicine, Stanford, CA 94305, USA; The Ohio State University Wexner Medical Center and OSU Comprehensive Cancer Center, Columbus, OH 43210, USA; Department of Cell and Tissue Biology, University of California San Francisco, San Francisco, CA 94143, USA; Department of Chemical and Systems Biology, Stanford University School of Medicine, Stanford, CA 94305, USA; Department of Radiation Oncology, Stanford School of Medicine, Stanford, CA 94305, USA

## Abstract

Adult salivary stem/progenitor cells (SSPC) have an intrinsic property to self-renew in order to maintain tissue architecture and homeostasis. Adult salivary glands have been documented to harbor SSPC, which have been shown to play a vital role in the regeneration of the glandular structures postradiation damage. We have previously demonstrated that activation of aldehyde dehydrogenase 3A1 (ALDH3A1) after radiation reduced aldehyde accumulation in SSPC, leading to less apoptosis and improved salivary function. We subsequently found that sustained pharmacological ALDH3A1 activation is critical to enhance regeneration of murine submandibular gland after radiation damage. Further investigation shows that ALDH3A1 function is crucial for SSPC self-renewal and survival even in the absence of radiation stress. Salivary glands from *Aldh3a1*^–/–^ mice have fewer acinar structures than wildtype mice. ALDH3A1 deletion or pharmacological inhibition in SSPC leads to a decrease in mitochondrial DNA copy number, lower expression of mitochondrial specific genes and proteins, structural abnormalities, lower membrane potential, and reduced cellular respiration. Loss or inhibition of ALDH3A1 also elevates ROS levels, depletes glutathione pool, and accumulates ALDH3A1 substrate 4-hydroxynonenal (4-HNE, a lipid peroxidation product), leading to decreased survival of murine SSPC that can be rescued by treatment with 4-HNE specific carbonyl scavengers. Our data indicate that ALDH3A1 activity protects mitochondrial function and is important for the regeneration activity of SSPC. This knowledge will help to guide our translational strategy of applying ALDH3A1 activators in the clinic to prevent radiation-related hyposalivation in head and neck cancer patients.

Significance Statement Radiation therapy in head and neck cancer patients are associated with severe side effects such as xerostomia/dry mouth. It occurs due to the radiation induced salivary gland atrophy and dysfunction. Our goal is to identify ways to protect salivary gland from radiation damage. We previously reported a role of a specific enzyme Aldehyde dehydrogenase 3A1 (ALDH3A1) activator Alda-341 in improving salivary gland function after radiation in mice by decreasing radiation associate aldehyde load. Here, we report that ALDH3A1 activity is also important for mitochondrial function to influence survival of salivary gland stem cells. This study bolsters the translational potential of the activator molecule to improve salivary gland function in patients.

## Introduction

Salivary glands function to produce and secrete saliva that aids in the digestion of food and maintenance of oral health. In humans, there are three pairs of major salivary gland namely parotid, submandibular, and sublingual that provide 90% of resting saliva volumes (1). Loss of function due to pathological atrophy of salivary glands (xerostomia) is a common complication associated with radiation therapy (RT) in head and neck cancer (HNC) patients. Xerostomia leads to difficulty in chewing, swallowing food, dental decay, weight loss, and overall poor quality of life (2). Because of their proximity to the draining lymph nodes, the submandibular glands (SMGs) are most likely to be damaged by radiation, including intensity modulated radiotherapy (IMRT) (3, 4). The only approved drug to prevent RT-related xerostomia is Amifostine, which is rarely used because of its significant side effects. Other interventions, such as pilocarpine for symptom relief, are minimally effective and require use for many years (5). This therapeutic gap has led us to focus on understanding salivary gland biology in order to protect them from radiation-induced damage (6–9).

Morphologically, SMGs are primarily composed of ductal and acinar cells. Acinar cells can be mucous or serous types that produce saliva, which is then moved through an organized ductal system into the oral cavity via the Wharton duct. Independent studies including lineage tracings or label retaining experiments have identified ductal stem cell markers, such as C-kit, Ascl3, Ck5, and Ck14 (10–15). Recently, the existence of self-renewing cells in the acinar compartment that could undergo self-duplication to regenerate and maintain homeostasis has also been described (16). Multiple reports investigating the response of salivary stem/progenitor cells (SSPC) to stressors using mouse salivary gland embryogenesis model, lineage tracing as well as adult murine salivary gland irradiation models have illustrated complex signaling mechanisms driving self-renewal, differentiation, and regeneration (17).

We previously identified a novel role of aldehyde dehydrogenase 3A1 (ALDH3A1) in facilitating recovery of salivary gland function in irradiated murine SMG models (9). ALDH3A1 is one of the 19 isoforms of the ALDH family that is primarily known for its detoxification function in corneal epithelial cells (18). We demonstrated that increasing ALDH3A1 activity using its specific activator, Alda-341 (d-limonene), protected murine SMG SSPC from RT-induced toxic aldehydes, leading to less SSPC cell deaths and improvement of salivary gland function. Intriguingly, we noted that sustained ALDH3A1 activation with Alda-341 long after clearance of RT-induced toxic aldehydes is critical to enhance regeneration of murine SMG after RT damage. Since *Aldh3a1* is one of the top enriched genes found in SSPC compared to non-SSPC from SMGs (6, 9), we hypothesize that it is also important for stem cell function and salivary gland development regardless of any stressor. Unexpectedly, we found that loss of *Aldh3a1* leads to mitochondrial dysfunction that can negatively affect self-renewal, differentiation, and survival of murine SSPC.

## Results

### Enhanced ALDH3A1 activity after irradiation is essential for SSPC survival and regeneration

**Fig. 1.**
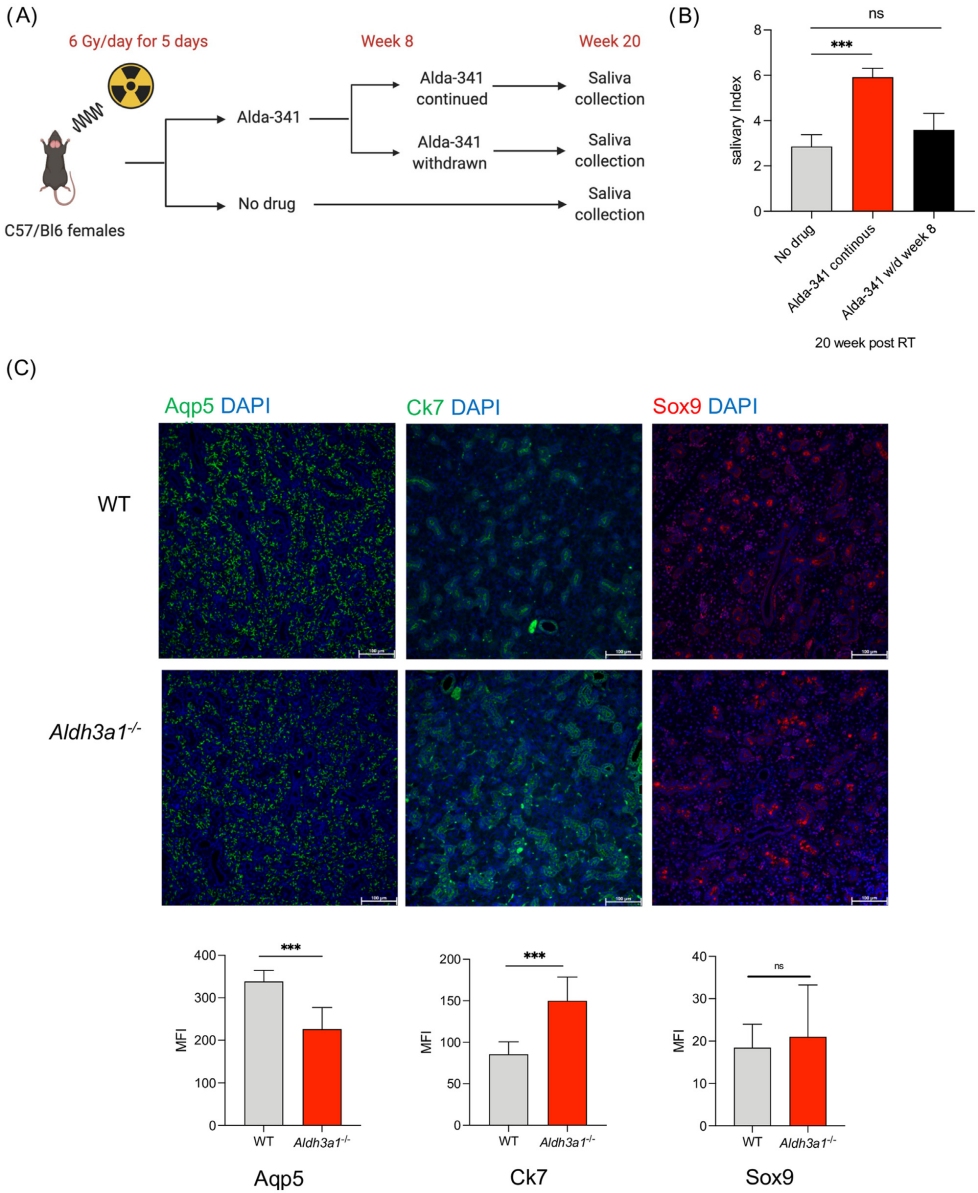
Sustained ALDH3A1 activity improves salivary function post-RT in mice and its genetic deletion alters SMG tissue morphology. (A) SMGs of mice were irradiated (30 Gy, 6 Gy x 5) followed by Alda-341 drug treatment for various time points up to 20 weeks. (B) Salivary index (saliva volumes/gm of body weight) of mice from different treatment groups at week 20 post 30 Gy (6 Gy/day) radiation to the salivary glands (30 Gy + Alda-341 continuous for 20 weeks, 30Gy + with Alda-341 withdrawn at week 8, 30 Gy + no drug, and *N* = 7 to 8 per group. (C) Immunofluorescent staining of acinar (Aqp5) and ductal (Ck7, Sox9) markers in SMGs derived from WT and *Aldh3a1^–/–^* mice imaged at 200x total magnification and is quantified and represented as mean fluorescence intensity (MFI) in the lower panel (10 images per staining, *n* = 3 mice/group). Scale bar: 100 μM. One-way ANOVA with multiple comparison was used to calculate the p value for panel (B). Student's t test was used to determine *P-*value in panel (C). Error bars represent SD (*represents *P-*value < 0.05 and ***< 0.001).

We had previously demonstrated that irradiation of murine SMGs reduced saliva function substantially, which could be partially rescued by treatment of ALDH3A1 activator, Alda-341, through a reduction of the aldehyde load (9). If reduced aldehyde load from radiation is the main driver to mitigate RT damage and restore function, then short-term treatment with the activator should suffice in sustaining improved saliva function. To address this hypothesis, we designed an experiment where Alda-341 treatment was started immediately after RT (30 Gy in five fractions to mimic fractionated radiation in the clinic), continued for 8 weeks, then either stopped and observed or continued for a total of 20 weeks (Fig. 1A). We had previously reported that this fractionated radiation regimen to the SMGs decreased salivary output by ∼60% in irradiated mice compared to unirradiated control mice, and continuous treatment of Alda-341 for 20 weeks improved saliva production over the nondrug-treated irradiated group (9). Interestingly, early termination of Alda-341 at 8 weeks post-RT resulted in a significant decline in saliva function at week 20, down to the level of the nondrug treated irradiated group and much lower than that of the continuously treated group (Fig. 1B). These results suggest that ALDH3A1 activation up to 8 weeks is not sufficient to lead to sustained improvement of salivary function. Of note to determine whether sustained treatment would result in long-term benefit, we treated another group of mice for 18 weeks and observed them for 4 weeks. An 18-week treatment led to sustained improvement that did not decline to the nondrug-treated irradiated level with drug withdrawal (Figure S1A, Supplementary Material). Thus, we hypothesize that apart from reducing RT-induced aldehyde load, ALDH3A1 may play another role in the regeneration process, and sustained enzyme activation is a requisite to see maximum benefits. These findings, in addition to our previous observation of elevated ALDH3A1 expression in SSPC, lead to the hypothesis that ALDH3A1 may play a role in SSPC function and survival.

### *Aldh3a1* deficiency alters murine SMG tissue morphology in adults and branching morphogenesis of embryonic explants

We assessed differences in tissue morphology of the three major salivary glands: parotid, submandibular, and sublingual in naïve *Aldh3a1^–/–^* mice as compared to WT mice. In both parotid and SMGs, we initially noted more ductal and less acinar compartments in the *Aldh3a1^–/–^* as compared to the WT mice on H&E staining (Figure S1B, Supplementary Material). Using Ck7 as ductal and Aqp5 as acinar marker, we confirmed that *Aldh3a1^–/–^* SMGs had significantly fewer Aqp5^+^ acinar and more Ck7^+^ ductal cells compared to WT SMGs (Fig. 1C).

**Fig. 2.**
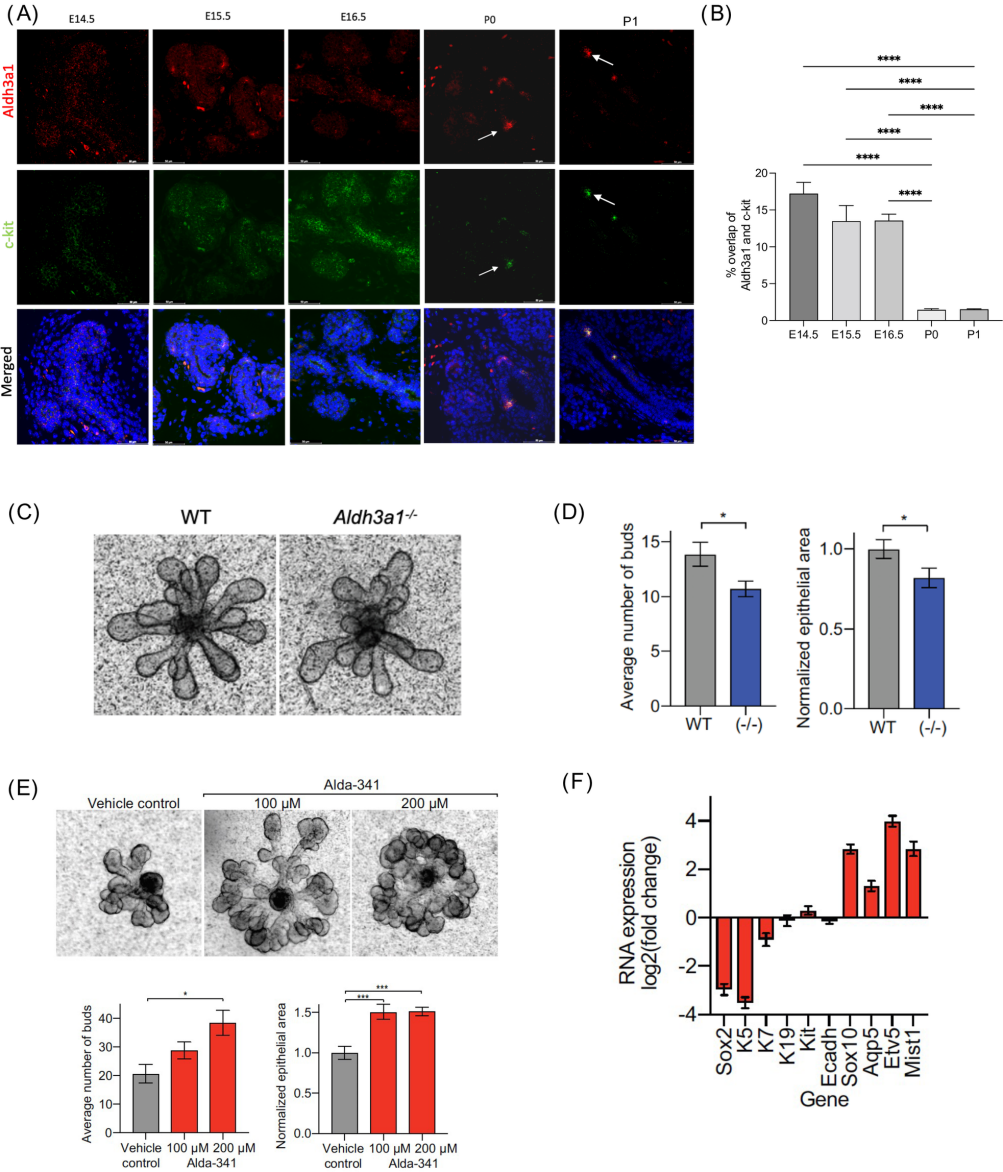
ALDH3A1 is expressed during development of embryonic salivary glands and is crucial for branching morphogenesis. (A) Representative images of RNAscope hybridization analyses of ALDH3A1 (red) and c-kit (green) in embryonic salivary glands isolated at various stages of development. Arrow points at enrichment of ALDH3A1 and c-kit expression in salivary glands of postnatal (p0 and p1) mice (5 to 6 epithelia/stage of development). Scale bar: 50 μM. (B) Quantification of percentage of cells showing overlap of c-kit and Aldh3a1 expression from RNAscope hybridization assay in different stages of development (eight independent field of view across multiple epithelia was used for analyses using QuPath). (C) Represents embryonic SMG epithelia (E13.5) from WT (left) and *Aldh3a1^–/–^* (right) mouse embryos cultured for 24 hours and imaged at 10x magnification. (D) Represents bud number counted from 17 WT and 16 *Aldh3a1^–/–^* epithelia (left panel) and average epithelial area quantified from 15 WT and 14 *Aldh3a1^–/–^* epithelia using Image J (NIH) and normalized to WT (right panel). (E) Representative images of E13.5 SMG epithelia from murine embryos that were treated with vehicle control, 100 μM, and 200 μM Alda-341 (left to right), cultured for 24 hours and imaged at 100x total magnification (*N* = 6 to 7 epithelia per group). Lower left panel: bud number was counted for each epithelium and averaged per group. Lower right panel: epithelial area was quantified using Image J (NIH) and normalized to vehicle control. (F) Reverse transcription quantitative PCR of RNA extracted from four epithelia per group. RNA expression of epithelia treated with 200 μM Alda-341 represented as a log2 fold change over RNA expression of epithelia treated with vehicle control. Error bars represent SD. One-way ANOVA with multiple comparisons was used to calculate *P-*value for panels (B) and (E). Student's t test was used to calculate the p value for panel (D) (*represents *P*-value < 0.05,  ***< 0.001, and ^****^< 0.0001).

To investigate the role of *Aldh3a1* in development, we analyzed the expression pattern of *Aldh3a1* mRNA in murine SMGs at E14.5, 15.5, 16.5, P0, and P1 using RNAscope. Murine *Aldh3a1* expression was ubiquitous and diffuse in early stage (E14.5 to 15.5) and became limited and concentrated in the stem cell compartment marked by *c-kit* expression during later stages of development of murine SMGs (P0 and P1) as shown in Fig. 2(A). Quantification of RNAscope data shows a significant decrease in percentage of cells coexpressing c-kit and Aldh3a1 at postnatal SMG as compared to embryonic stages (Fig. 2B). We also analyzed data from a published single cell RNA sequencing study done on murine embryonic and adult salivary glands to identify the cell populations that express *Aldh3a1* during development, postnatal, and adult murine SMG (19). UMAP plots show ubiquitous expression of *Aldh3a1* across different cell types at E12; the expression becomes restricted to specific cell populations at E14 and E16 (“Krt19^+^” and “Basal duct^+^ cell cluster”; Figure S2A, Supplementary Material). In the postnatal and adult glands, *Aldh3a1* mRNA is found to be enriched primarily in cell clusters of ductal origin (“krt19^+^ duct,” “Basal duct,” and “Gstt1^+^ ducts”; Figure S2B, Supplementary Material). In humans, ALDH3A1 was found to be expressed predominantly in the ductal compartment as represented by immunostaining images from three different patient-derived SMGs (Figure S3A, Supplementary Material). These results bolster our findings that *Aldh3a1* expression is predominantly limited to ductal cells during development, postnatal, and adult murine SMGs. Based on the morphological differences between *Aldh3a1^–/–^* and WT adult SMG, we hypothesized that ALDH3A1 may influence murine embryonic SMG differentiation. SMGs isolated from E13.5 *Aldh3a1^–/–^* and WT mice were carefully dissected to remove the epithelium from the mesenchyme; the epithelial rudiments branch to form end buds when grown for 24 hours as described previously (11). We observed that *Aldh3a1^–/–^* murine embryonic glands had fewer end buds with associated smaller total epithelial area as compared to WT glands (Fig. 2C and D). We then assessed the effect of activating ALDH3A1 with an activator Alda-341 (d-limonene) on branching morphogenesis of E13.5 murine SMGs and observed a significant dose-dependent increase in branching with more end buds in the Alda-341 treated group as compared to controls (Fig.   2E). In the Alda-341-treated group, stem cell *c-kit* expression was found to be limited in the end buds as compared to the control (Figure S3B, Supplementary Material). Quantitative PCR analyses revealed that the phenotypic difference after Alda-341 treatment appears to be orchestrated by increased expression of genes regulating differentiation (*Aqp5, Etv5*,and*Mist1*) and a decreased expression of stemness-related genes (*Sox2*and*Ck5*; Fig. 2F).

### ALDH3A1 activity is essential for self-renewal of murine and human SSPC

**Fig. 3.**
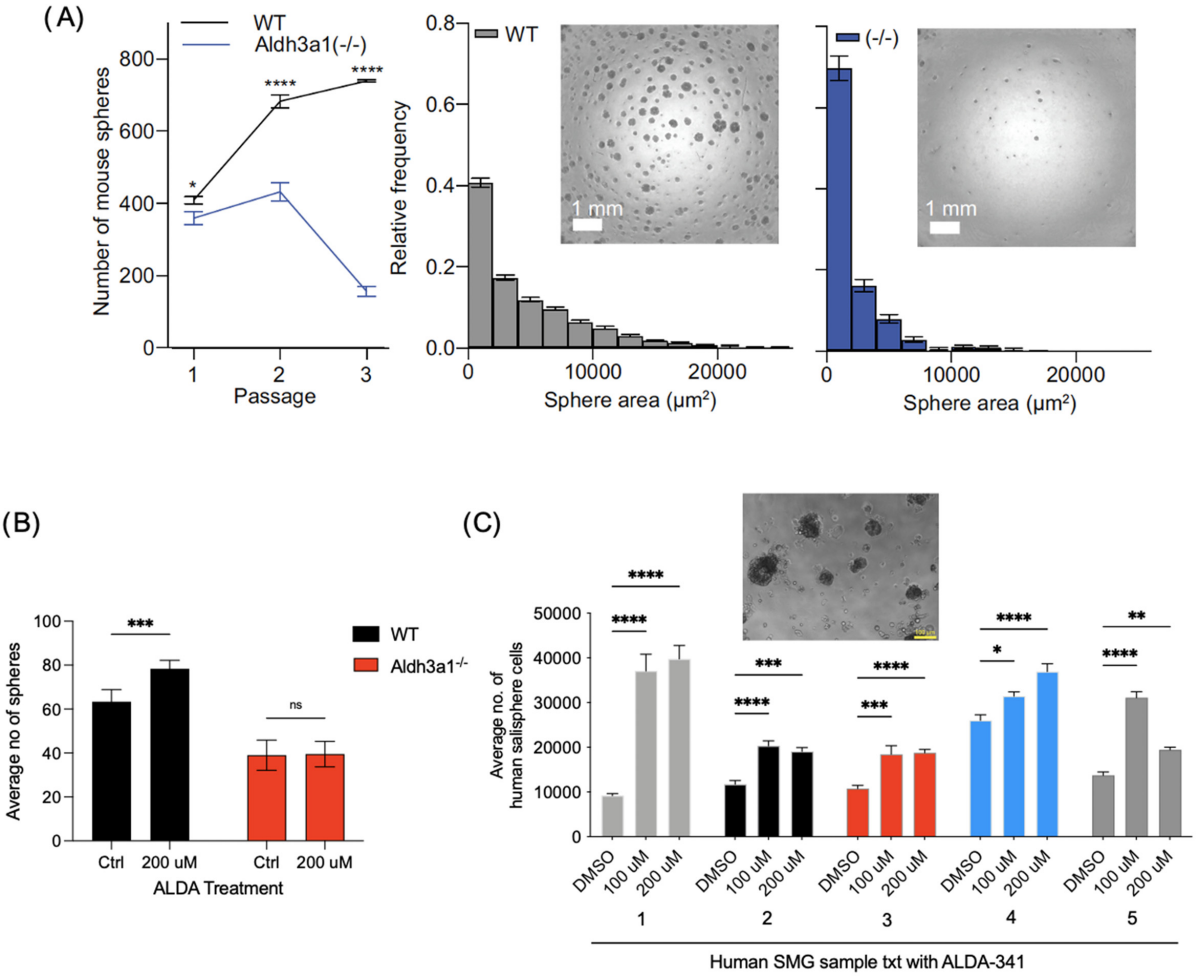
ALDH3A1 deficiency impacts self-renewal of murine and human patient derived SSPC. (A) Left panel shows the graph of the average number of spheres per well at day 7 for each passage by imaging each well and quantifying by Image J (NIH). Cells were passaged every 7 d for three passages (six replicates per group). Middle and right panel reflect the size of the spheres by quantifying the average frequency for the different sphere areas in WT and *Aldh3a1^–/-^ SSPC*. Representative images of spheres from WT and *Aldh3a1^–/–^* SSPC are also shown. (B) Number of spheres from murine WT and *Aldh3a1^–/–^* SSPC at day 7 post-treatment with 200 μM Alda-341 or vehicle control. Sphere number per well was quantified by Image J (NIH), (six replicates per group). (C) Average number of human salivary cells (dissociated from embedded human salivary spheres in matrigel) at day 7 in the presence of vehicle control, 100 or 200 μM Alda-341 (*n* = 5 patients, 3 technical replicates). Representative image of human salivary gland spheres is also shown. Error bars represent SD. Student's t test was used to calculate the *P-*value for panels (B) and (C). Two-way ANOVA with multiple comparisons was used to estimate *P-*value for panel (D) (*represents *P*-value < 0.05, **< 0.01, ***< 0.001, and ^****^< 0.0001).

To determine if ALDH3A1 function is crucial for the growth properties of murine SSPC, we investigated the effect of ALDH3A1 deficiency in self-renewal *in vitro*. Primary dissociated cells from murine salivary gland derived from age and gender matched WT and *Aldh3a1^–/–^* mice were FACS sorted for cells with EpCAM/CD24 high expression, which has previously been shown to be enriched for SSPC (7). These cells were then grown on a layer of growth factor reduced (GFR) matrigel supplemented with stem cell growth media (Figure S3C, Supplementary Material). By day 7, *Aldh3a1^–/–^* SSPC gave rise to significantly fewer spheres as compared to WT, which was consistent across multiple passages (Fig. 3A). To test the reverse, we treated murine SSPC, either WT or *Aldh3a1^–/–^*, with the ALDH3A1 activator Alda-341. Treatment with Alda-341 significantly increased the number of spheres only in the WT but not in *Aldh3a1^–/–^* group compared to vehicle treated controls (Fig. 3B). These data indicated that ALDH3A1 activation increased sphere formation and the effect of Alda-341 is specific to its activation of ALDH3A1 enzyme. Increased self-renewal of SSPC by Alda-341 was also extended to human samples; treatment of SSPC from five patient-derived SMG cultures with two concentrations of Alda-341 similarly resulted in increased sphere formation compared to vehicle control (Fig. 3C).

### Gene expression analyses identified dysregulated mitochondrial gene expression in *Aldh3a1^–/–^* SSPC

**Fig. 4.**
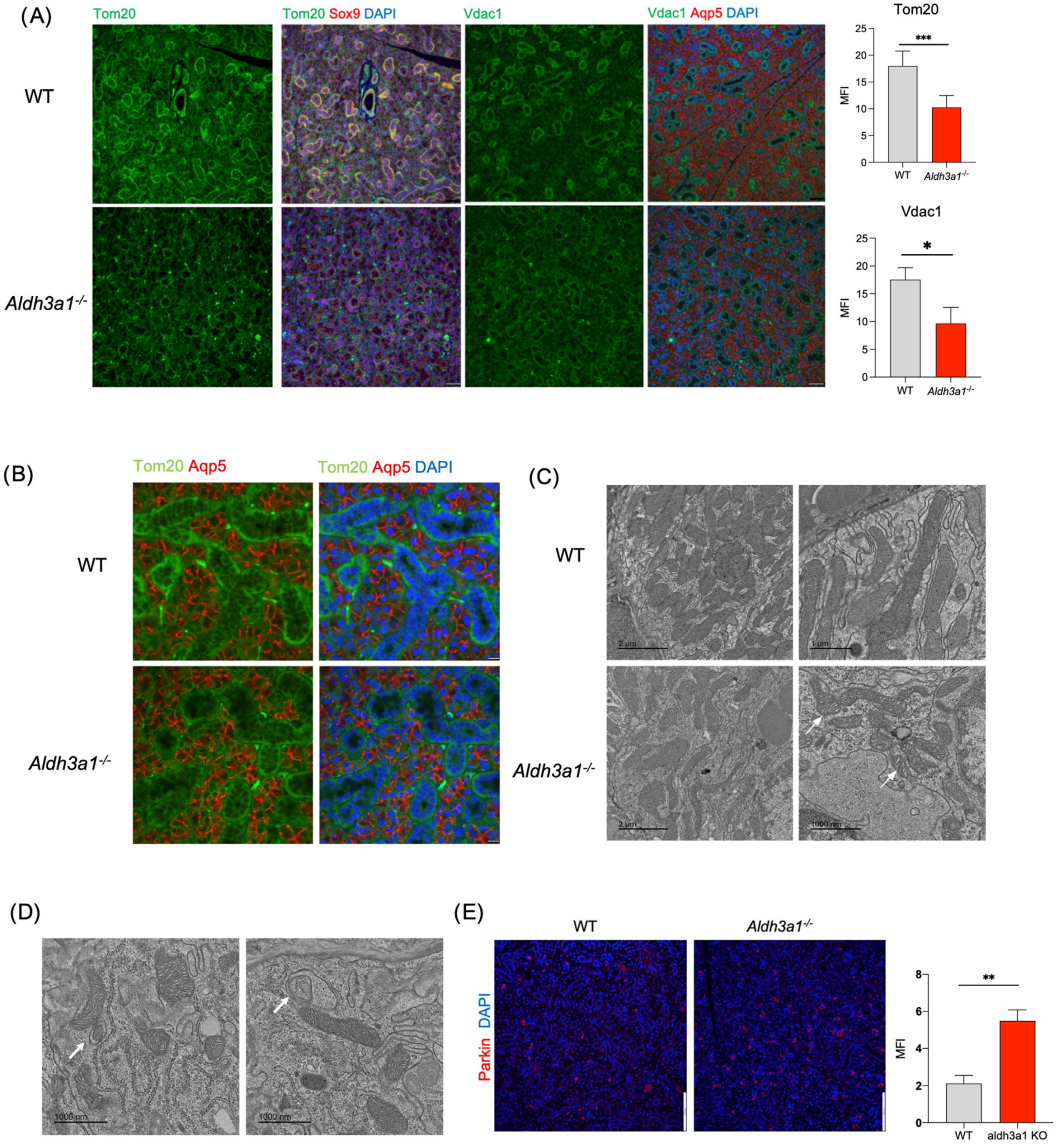
*Aldh3a1* deficient SMG have lower protein expression, lower mitochondrial abundance, altered morphology, and increased mitophagy. (A) Representative Immunostaining images of mitochondrial (Tom20 and Vdac1), ductal (Sox9), and acinar (Aqp5) markers in WT and *Aldh3a1^–/–^* SMGs. Scale bar: 50 μM (10 images per staining, *n* = 3/group). Right panel shows quantification of Tom20 and Vdac1 staining in WT and *Aldh3a1^–/–^* SMG. A total of 10 random field of view used for quantification per using ImageJ and represented as the Mean of Fluorescence intensity (MFI). (B) High magnification immunostaining image of WT and *Aldh3a1^–/–^* SMGs showing mitochondrial marker Tom20 and Acinar marker Aqp5. (C) TEM image of ductal cells in SMGs derived from WT and *Aldh3a1^–/–^* showing more mitochondrial abundance and better ultrastructure in WT cells (*n* = 3/group, scale bar = 2 μM left panel, and 1 μM right panel). (D) Representative TEM images of *Aldh3a1^–/–^* ductal cells in SMG showing mitophagy (arrow). (E) Immunostaining analyses of mitophagy marker, Parkin, in WT and *Aldh3a1^–/–^* SMGs imaged at 200x total magnification and is quantified and represented in the right panel (*n* = 3/group and scale bar: 130 uM). Error bars represent SD. Student's t test was used to calculate the *P*-value (*represents *P-*value < 0.05, **< 0.01, and ***< 0.001).

To identify molecular pathways impacted by *Aldh3a1* deficiency, we performed RNA-seq analyses on freshly isolated WT and *Aldh3a1^–/–^* murine SSPC (*n* = 3/group). In total, 175 genes showed a significant log fold change with an FDR < 0.1 (Figure S3D, Supplementary Material).  Gene Ontology analysis of these 175 genes using MetaCORE identified mitochondrial pathways to be significantly different in the *Aldh3a1^–/–^* SSPC as compared to WT (Figure S3E, Supplementary Material). A total of nine mitochondrial encoded genes were downregulated in the *Aldh3a1^–/–^* cells as compared to WT cells (Figure S4A, Supplementary Material). Q-PCR of mitochondrial genes in freshly isolated murine WT and *Aldh3a1^–/–^* SSPC showed an overall trend of reduced gene expression, with a statistically significant difference noted in some candidate genes (mt-Nd2, mt-Nd3, and mt-Nd4; Figure S4B, Supplementary Material). Mitochondrial DNA copy number was also lower in *Aldh3a1^–/–^* SSPC compared to WT, as reflected by the lower level of mitochondrial coded genes, *Cycs, Cox3*, and *16rs*, normalized to nuclear coded gene, *β-globin* (Figure S4C, Supplementary Material). Immunostaining experiments showed reduced expression of mitochondrial markers Tom20 and VDAC1 in *Aldh3a1^–/–^* SMGs as compared to WT SMGs (Fig. 4A and B). These differences in mitochondrial marker expression were also observed in primary spheres derived from *Aldh3a1^–/–^* and WT murine SSPC when analyzed by Immunostaining (Figure S4D, Supplementary Material). Ultrastructure analyses of ductal cells using transmission electron microscopy (TEM) revealed reduced abundance of mitochondria and deformities in cristae and intramembranous space in of *Aldh3a1^–/–^* SMGs (Fig. 4C). Please note that mitochondria are much less abundance and identifiable in acinar compared to ductal cells because of the rich endoplasmic reticulum network in acinar cells, obscuring other organelles (Figure S4E, Supplementary Material). We also observed more mitochondria in ductal cells undergoing mitophagy in *Aldh3a1^–/–^* SMGs using TEM (Fig. 4D). Mitophagy marker, Parkin1, was increased in *Aldh3a1^–/–^* SMGs as compared to WT, suggesting increased mitophagy (Fig. 4E). Moreover, activation of ALDH3A1 by Alda-341 (d-limonene) treatment in mice upregulated mitochondrial related genes in WT SSPC as demonstrated by our independent gene expression analyses (Figure S5A and B, Supplementary Material). These results suggest a novel role of the cytosolic ALDH3A1 in influencing mitochondrial content, structure, protein expression, and dynamics in murine salivary glands.

### ALDH3A1 impacts mitochondrial function of murine salivary gland cells

**Fig. 5.**
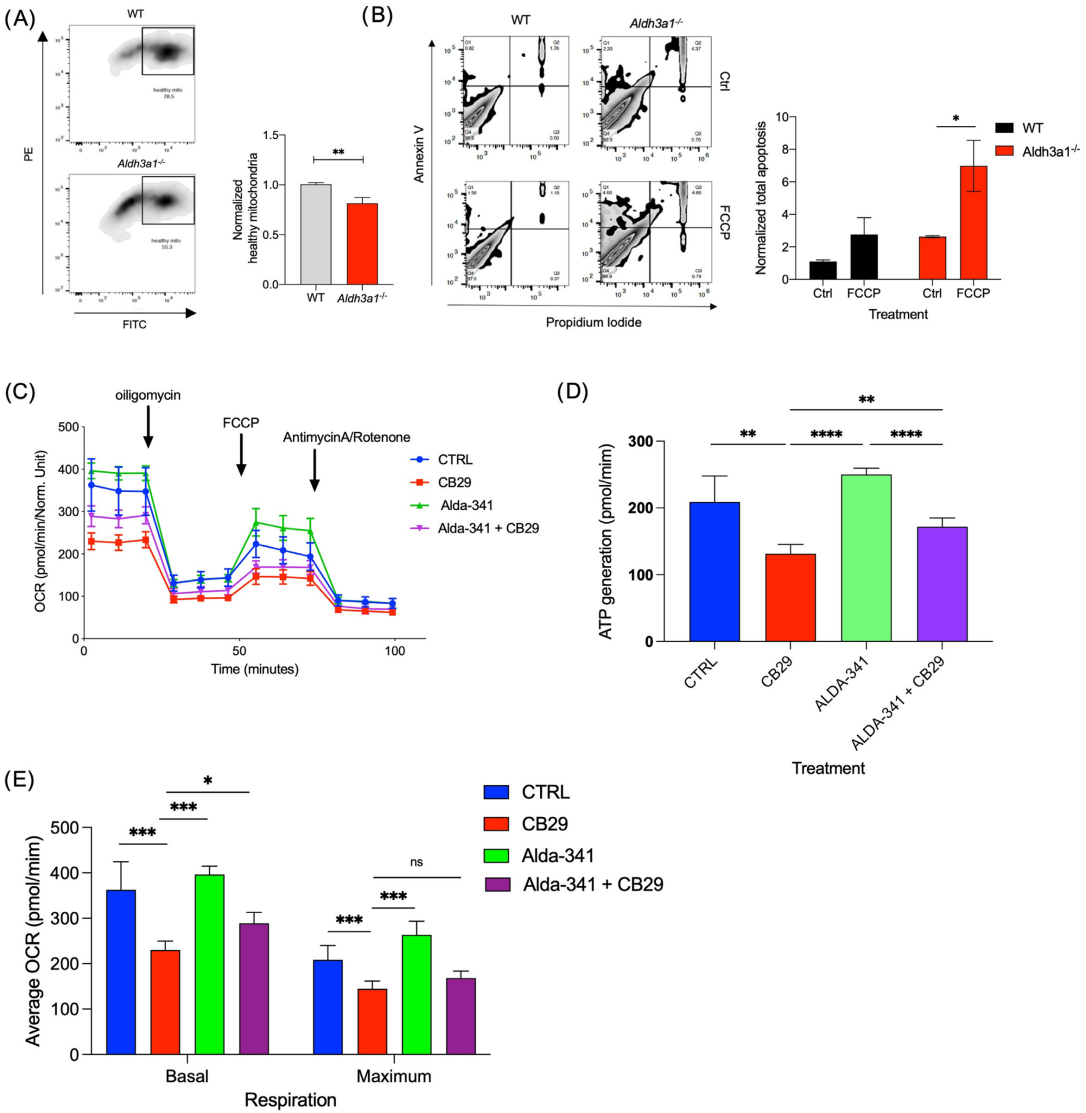
*Aldh3a1* deficient SSPC display attributes of dysregulated mitochondrial function as compared to WT. (A) JC-1 assay analysis of WT and *Aldh3a1^–/–^* murine SSPC showing proportions of PE-high/FITC-high cells with mitochondria that have intact membrane potential. This is quantified and represented in the graph in right panel (*n* = 3 and 2 technical replicates). (B) Quadrant plots showing Annexin-V/PI staining of WT and *Aldh3a1^–/–^* murine SSPC 24 hours post-treatment with mitochondrial inhibitor FCCP; this is quantified and represented in Graph in right panel (*n* = 3 with technical duplicates). The data is normalized to % apoptosis in the WT group. (C) Average OCR over time of mSGc cells treated with vehicle, 10 uM ALDH3A1 inhibitor CB29, 100 uM Alda-341, and combination of both. (D) Average ATP production in various treatment groups. (*n* = 2 with 4 technical replicates). (E) Average basal and maximum respiration in various treatment groups. Error bars represent SD. Student's t test was used to calculate the *P-*value for panel (B) and one-way ANOVA with multiple comparisons was used for panels (D) and (E) (*represents *P-*value < 0.05, **< 0.01, ***< 0.001, and ^****^< 0.0001).

We assessed alterations in mitochondrial functional attributes in terms of membrane potential, sensitivity to mitochondrial inhibitor and respiration in murine salivary cells with decreased or no ALDH3A1 activity in comparison to controls. Loss of membrane potential in mitochondria is linked to mitochondrial dysfunction. Using the JC-1 assay, we found that *Aldh3a1^–/–^* SSPC had a significantly lower average membrane potential as compared to WT SSPC (Fig. 5A). *Aldh3a1^–/–^* SSPC were also two-folds more sensitive to a mitochondrial uncoupler, FCCP, as represented by a significantly higher percentage of positive cells undergoing apoptosis at 24 hours after treatment as compared to WT SSPC (Fig. 5B).

Differences in mitochondrial integrity and protein expression can translate to deficiency in mitochondrial primary function, which is cellular respiration and ATP production. We could not employ our sphere model for this experiment due to constraints of growth requirements and adherent conditions. To overcome this, we used a normal murine salivary gland cell line (mSGc) as an alternate model. mSGc can be maintained for multiple passages without a loss of proliferation potential, readily form 3D-spheroids and importantly express a panel of well-established salivary gland epithelial cell markers (20). We used a previously reported specific inhibitor of ALDH3A1 activity, CB29 (21), to inhibit ALDH3A1 activity in the cell line model. The EC50 of the inhibitory effect of CB29 to ALDH3A1 was determined by a dose–response study (Figure S6A and B, Supplementary Material). To test the effect of ALDH3A1 inhibition on mitochondrial respiration, we treated mSGc cells with CB29 and measured the basal and maximum oxygen consumption rate (OCR) as well as ATP production using the Seahorse XF analyzer 24 hours post-treatment. We found a significant decrease in ATP production, basal and maximum respiration in the CB29 treated cells as compared to vehicle control. In contrast, cotreatment of CB29 plus the activator Alda-341 partially rescued the effects of the inhibitor by improving ATP production and basal respiration as compared to CB29 treatment alone (Fig. 5C–E). CB29 treatment of WT SSPC also led to more apoptosis with associated decreased sphere formation as compared to vehicle control (Figure S6C and D, Supplementary Material). To complement the inhibitor experiments, we also used siRNA approach against *Aldh3a1* and confirmed that reduced *Aldh3a1* expression led to increased apoptosis and decreased sphere formation efficiency in mSGc cells (Figure S6D–F, Supplementary Material).

### Levels of ROS, glutathione, and ALDH3A1 substrate 4-HNE influences survival of *Aldh3a1^–/–^* SSPC

**Fig. 6.**
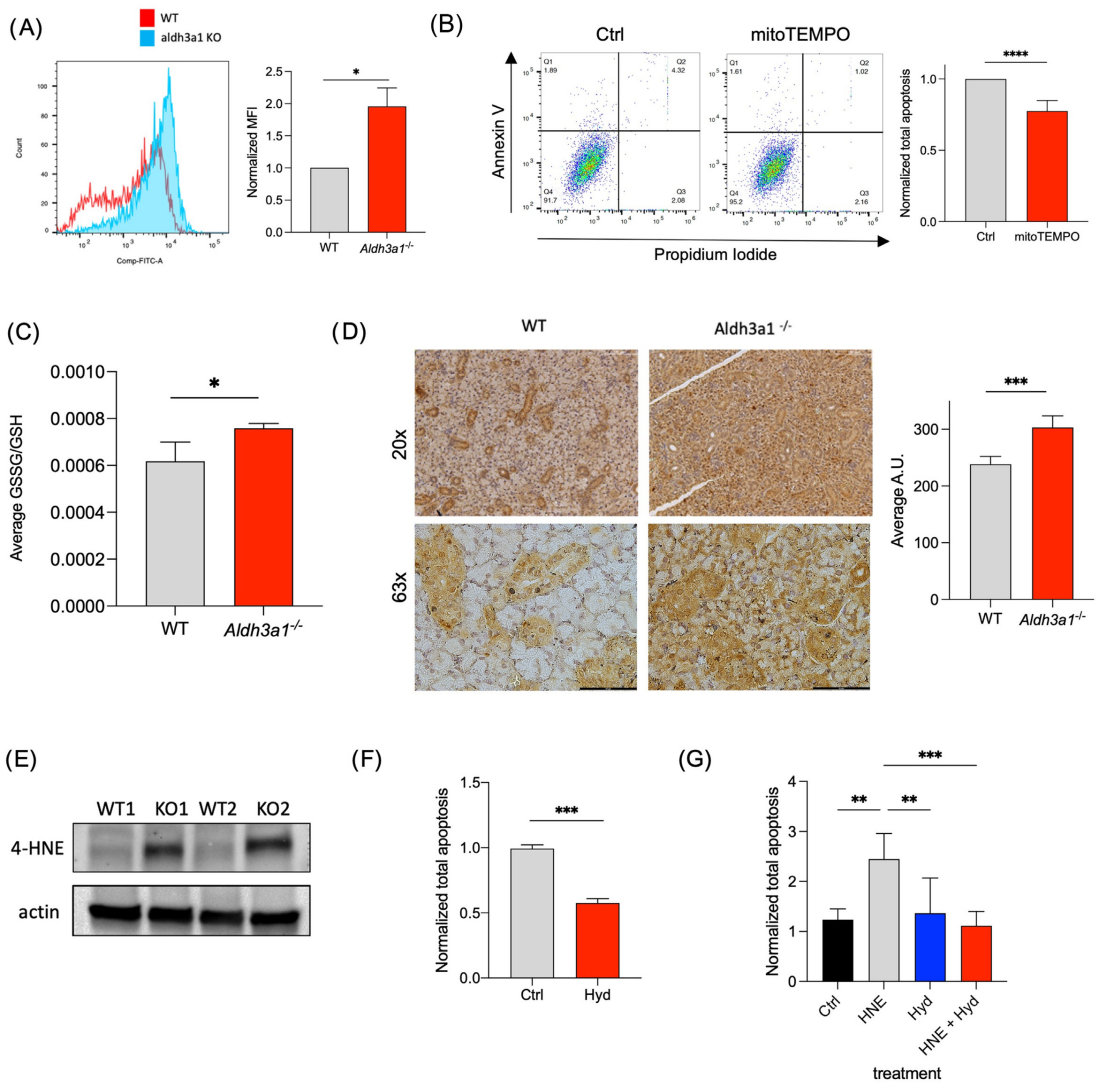
Accumulation of ROS and substrate 4-HNE in *Aldh3a1* deficient SSPC drives poor survival. (A) Histogram plots of ROS levels determined by FACS analyses in WT and *Aldh3a1^–/–^* murine SSPC; this is quantified and represented in the right panel (*n* = 3 and 2 technical replicates). (B) Bar graph showing % apoptosis of *Aldh3a1^–/–^* murine SSPC 24 hours normalized to vehicle control post-treatment with100 nM mito-TEMPO. (*n* = 2 and 3 technical replicates). (C) Carbon labeling experiments demonstrate differences in glutathione turnover in WT and *Aldh3a1^–/–^* SSPC represented as the ratio of absolute abundance of GSSG and GSH. (D) Immunostaining and (E) Western blot analyses of 4-HNE in WT and *Aldh3a1^–/–^* SMGs (*n* = 3/group for IHC, *n* = 2 for western blotting). Scale bar: 100 μM. (E) Average % apoptosis in *Aldh3a1^–/–^* SSPC 24 hours post-treatment with 10 uM of hydralazine normalized to control. (*n* = 3 and 3 technical replicates). (F) Average % apoptosis in WT SSPC 24 hours post-treatment with 10 uM 4-HNE, 10 uM of hydralazine or the combination with 4-HNE and hydralazine normalized to control (*n* = 2 and 3 technical replicates). Error bars represent SD. Student's t test was used to calculate the *P-*value for panels (A) to (D) and (F). One-way ANOVA with multiple comparisons was used for panel G (*represents *P-*value < 0.05, **< 0.01, and ***< 0.001).

Increased ROS levels have been associated with mitochondrial dysfunction. In the murine SSPC model, ROS levels were significantly higher in the *Aldh3a1^–/–^* SSPC as compared to WT SSPC (Fig. 6A). We have previously found that *Aldh3a1^–/–^* SSPC have higher basal apoptosis as compared to WT cells (9). To demonstrate that ROS accumulation impacts survival, we tested the effect of antioxidant treatment on survival of murine *Aldh3a1^–/–^* SSPC. Treatment of *Aldh3a1^–/–^* cells for 24 hours with mito-TEMPO, a mitochondria-specific ROS scavenging molecule, significantly improved *Aldh3a1^–/–^* SSPC survival as compared to vehicle control (Fig. 6B). Glutathione plays antioxidative role against elevated ROS. ALDH3A1 helps to convert oxidized glutathione (GSSG) to its reduced form (GSH) (22). As expected, in the absence of ALDH3A1, we observed an increased GSSG–GSH ratio in murine *Aldh3a1^–/–^* SSPC by LC/MS analyses (Fig. 6C), which can contribute to oxidative stress. To test if GSH depletion has an independent effect on poor survival of *Aldh3a1^–/–^* SSPC, we supplemented WT and *Aldh3a1^–/–^* SSPC with *N*-Acetyl-L-cysteine (NAC, a cysteine precursor that can boost GSH levels) and assessed sphere formation at day 7. NAC treatment led to a significant increase in sphere formation for *Aldh3a1^–/–^* SSPC compared to vehicle control but no difference for WT SSPC (Figure S7A, Supplementary Material).

One of the main substrates of ALDH3A1 is 4-hydoxynonenal (4-HNE) which is a product of lipid peroxidation (LPO).We observed increased accumulation of 4-HNE in *Aldh3a1^–/–^* SMG as compared to WT SMG via immunostaining and western blotting (Fig. 6D and E). Hydrazine derivatives have been reported to rescue the effects of 4-HNE accumulation in smooth muscle cells (23). As expected, hydralazine treatment on *Aldh3a1^–/–^* SSPC for 24 hours significantly decreased the proportion of cells undergoing apoptosis and improved survival (Fig. 6F).  To confirm if the hydrazine acts like a scavenger for 4-HNE, we exposed WT SSPC for 24 hours with 4-HNE alone, hydralazine alone, or the two together and assessed apoptosis. There was an induction of cell death with 4-HNE treatment alone, which was rescued upon cotreatment of hydralazine (Fig. 6G). Overall, our data suggests ALDH3A1 deficiency leads to increased accumulation of ROS, its byproduct 4-HNE, and decreased GSH that contributes to poor survival of SSPC.

## Discussion

Gene expression profiling of murine SSPC had helped us to identify and validate GDNF and ALDH3A1 as crucial determinants of salivary gland function and repair upon radiation stress (7,8). ALDH3A1-deficient mice display poorer saliva function after RT as compared to WT. Treatment with the ALDH3A1 activator, Alda-341 (d-limonene), improved function of SMGs by reducing the acute RT-induced aldehyde load and promoting SSPC survival (9). However, our data showing that long-term activation of ALDH3A1 (upto 18 to 20 weeks) with Alda341 (d-limonene) is needed for long-term maintenance of improved saliva function suggest that ALDH3A1 plays a more sustained role in SSPC renewal rather than just acute clearance of radiation-induced aldehyde. This led us to explore the effect of ALDH3A1 in SSPC phenotype using both embryonic and salisphere models.

We found that ALDH3A1 is important for self-renewal, survival, and differentiation of murine SSPC beyond acute radiation stress. Genetic deletion of *Aldh3a1* results in morphological differences in murine SMG that can be attributed to abnormal development patterns. Branching morphogenesis of murine embryonic SMGs is an excellent model to study the effect of cell specific ablation of factors important for differentiation or development (12). Reduced branching and number of end buds in *Aldh3a1* deficient explants suggests its role in acinar cell development. These findings resonate with other studies; for example, SOX2 was identified as a marker of SMG progenitor cells that are capable of giving rise to acinar cells but not ductal cells (24). Indeed, differences observed in embryonic morphogenesis of fetal explants can explain why we see reduced acinar compartment in adult SMGs of KO mice as compared to WT. However, the potential role of ALDH3A1 in the context of adult salivary stem cell differentiation is yet to be explored.

ALDH3A1 activity also promoted self-renewal of murine and human SSPC. Self-renewal is a characteristic attribute of adult stem cells, which upon division gives rise to a differentiated daughter cell and a stem cell. Sphere formation assay from isolated murine SSPC is an *in vitro* technique to assess changes in self-renewal upon treatment or intervention (25). As sphere formation is a balance of proliferation and survival, we predict that ALDH3A1 deficiency affects SSPC survival, thus shifting the overall balance and reducing sphere formation.

Our in-depth gene expression analyses identifies a strong link between *Aldh3a1* deficiency and reduced mitochondrial gene expression and function in SSPC. A survey of the literature showed only two studies that reported a relationship between ALDH3A1 and mitochondrial function. In a yeast model, a homolog of ALDH3A1 plays a critical role in the synthesis of a precursor molecule of coenzyme Q, an important carrier of the mitochondrial electron transport chain (ETC) (26). In gastric cancer, inhibiting ALDH3A1 impairs mitochondrial activity due to reduced beta oxidation of lipids and acetyl co-A flux for TCA cycle (27), which was also demonstrated by our LC/MS analyses (Figure S7B, Supplementary Material). These cumulative findings strongly demonstrate that activation of the cytosolic ALDH3A1 is needed to support mitochondrial function in SSPCs under basal, nonstressed conditions. Although we primarily focus on ductal cells due to the enrichment of *Aldh3a1* expression (single cell RNAseq) and mitochondria number (TEM) in the ductal compartment, we cannot exclude the possibility that the same process may also be occurring in acinar cells, especially self-renewing ones. As shown in the magnified view, increased 4-HNE level is seen in both ductal and acinar cells with *Aldh3a*1 deletion despite the low expression of this gene in the acinar compartment. Future work is needed to determine the role of ALDH3A1 in acinar cells.

Mechanistically, our data suggest a vicious cycle between loss of ALDH3A1 activity and mitochondrial function: increased ROS levels in *Aldh3a1^–/–^* SSPC leads to higher oxidative stress, reduced glutathione turnover, increased 4-HNE accumulation and mitochondrial damage, and reduced survival (Figure S8A, Supplementary Material). This is consistent with prior data showing that irradiation to the saliva glands leads to an increase in mitochondrial ROS and loss of membrane potential, resulting in caspase 3 activation and subsequent apoptosis, that can be improved by the treatment of mitochondria specific scavenger mito-TEMPO (28,29). Mitochondria are the primary source of ROS, which is partially converted to toxic products such as 4-Hydroxynonenal (4-HNE) via LPO (30,31). ALDH3A1 inactivates 4-HNE, as well as replenishes reduced glutathione pool to maintain homeostasis and viability (18, 26). GSH depletion has been attributed to the activation of apoptosis via post-translational modifications, thus providing a parallel mechanism that contribute to poor survival of SSPC deficient of ALDH3A1 (32). The loss or decrease activity of ALDH3A1 leads to accumulation of 4-HNE, which in turn causes protein adduct formation and inactivation, mitochondrial dysfunction, increased ROS accumulation, and increased apoptosis in cells (33, 34). 4-HNE accumulation can induce cell death via extrinsic or intrinsic apoptotic pathways by causing DNA damage in cells (35, 36). In our model, we found higher gammaH2AX foci as well as DNA damage response gene expression (*p21* and *Bax*) in *Aldh3a1^–/-^* SMG and SSPC, respectively as compared to WT (Figure S8B, Supplementary Material). These findings is consistent with the previously identified role of ALDH3A1 to protect against DNA damage in human corneal and bronchial epithelial cells (37, 38).

Physiologically, adult stem cells are found in hypoxic niche; thus, they normally experience low ROS levels (39). In such hypoxic environment, adult stem cells prefer glycolysis and fatty acid oxidation as metabolic drivers. Based on previously identified role (27), we can postulate that ALDH3A1 function is crucial for efficient FAO in these cells. Adult stem cell niche are also sensitive to ROS produced by radiation that can prime them for differentiation and apoptosis (40,41). Increased ALDH3A1 activity together with replenished GSH pool operate as oxidative stress responses to protect adult stem cells from ROS damage. Stress such as radiation, increases ROS levels and impairs their ability to self-renew and proliferate long term, preventing regeneration of salivary gland even with termination of the radiation insult (42,43). Our data showed that sustained activation of ALDH3A1 can protect the cells from oxidative damage over time, leading to long-term survival of SSPC and sustained improvement of salivary function. Future work will explore how ALDH3A1 activity can influence other effects such as chronic inflammation postradiation that poses a greater risk for tissue regeneration (44). Overall, our data helped to guide the duration of d-limonene treatment in a phase I trial that has just been activated in HNC patients (NCT04392622). In this trial, d-limonene is administered during and up to 20 weeks after definitive chemoradiotherapy for salivary gland protection from radiation damage.

In summary, our data indicate that ALDH3A1 activity is required to support embryonic differentiation and self-renewal function of adult salivary stem cells, at least in part, by maintaining mitochondrial number and functions. It explains the differences in tissue architecture when this enzyme is deleted and supports the continuing use of ALDH3A1 activators after the end of radiation to assist stem cell renewal and possibly differentiation in that setting.

## Methods

### Animals used

C57BL/6 mice (7 to 10 weeks) were purchased from Jackson Laboratories (Bar Harbor, ME). C57BL/6J *Aldh3a1^–/–^* mice were obtained from the laboratory of Vasilis Vasiliou at Yale School of Public Health, New Haven, CT (45). Experiments were done with 8 to 12 weeks old female mice. All the protocols were approved by The Administrative Panel on Laboratory Animal Care (APLAC) at Stanford University, Stanford, CA. All the experiments involving animals were done in adherence to the NIH Guide for the Care of and Use of Laboratory Animals. For our analyses, we used female mice because murine SMGs display sexual dimorphism and female SMGs show closer resemblance to humans (46).

### Salivary gland isolation and culture from mouse and human SMGs

Murine salivary glands were dissected and isolated from euthanized mice as described previously (8). Please see Supplemental methods for details.

### RNA sequencing

RNA sequencing experiment was done as previously described (9). Please see Supplemental methods for details.

### Single-cell RNA-Seq data analyses

Ready to use SEURAT objects for Embryonic and Postnatal SMG integrated datasets were retrieved from figshare as indicated in the original study (19). The code used for the analyses is provided in Data S1 (Supplementary Material).

### RT-PCR

RNA was isolated from whole tissue or SSPC using RNAqueous Micro Kit (Ambion, Austin, TX). Total RNA samples were DNase-treated (Ambion) before complementary DNA (cDNA) synthesis using SuperScript reagents according to manufacturer's protocol (Invitrogen, Waltham, MA). SYBRgreen RT-qPCR was performed using cDNA and primers used before (47) or designed using Primer3 and Beacon Designer software or found using PrimerBank (http://pga.mgh.harvard.edu/primerbank/). Gene expression was normalized to the housekeeping gene S29 (Rps29) or actin.

### Embryo salivary gland dissection and branching morphogenesis assay

Salivary glands were dissected out during various stages of murine embryos development as described previously (48). E13.5 salivary glands were used for the branching morphogenesis assay. Please refer to Supplemental methods for more details.

### EM imaging

Salivary glands from WT and *Aldh3a1^–/–^*  mice were dissected and used for TEM. Please refer to Supplemental methods for details.

### Mitochondria respiration

A total of 10,000 cells were seeded in Agilent (Santa Clara, CA) Seahorse XF96 cell culture microplates. Next day, cells were treated with vehicle control, CB29 (20 μM), Alda-341 (100 μM), and combination of the two drugs. After 24 hours of incubation, media was replaced, and cells were maintained at 37°C. Extracellular flux assay to measure mitochondrial respiration and ATP production was carried out as recommended by manufacturer's protocol (Agilent). SRB assay (49) was used to quantify cell concentration in samples for normalization of data.

### Mitochondrial isolation and copy number analyses

Mitochondrial DNA was isolated from a modified version of a protocol (50). Cells were scraped using RIPA lysis buffer and incubated with 5 μl of Proteinase K (Invitrogen) for 55°C for 3 hours. Samples were sonicated briefly to shear the DNA. The debris was removed from spinning down the samples at 8,000 *g* for 15 min. To the supernatant, equal volume of phenol/chloroform/Isoamyl alcohol mixture (25:4:1) was added. The samples were mixed well by vigorous shaking followed by centrifugation at 8,000 *g* for 15 min. The clear upper layer was collected, added to equal volume of chloroform/isoamyl alcohol and mixed well. The samples were spun down and the upper layer was transferred to a fresh tube, to which 40 μl of 3M sodium acetate and 440 μl of Isopropanol were added. Samples were incubated at −20°C for 10 min to facilitate DNA precipitation. Samples were spun again to pellet the precipitated DNA. Supernatant was discarded and the pellet was washed with 70% alcohol. The supernatant was discarded, and the dried pellet was resuspended in molecular grade water (Invitrogen). For copy number analyses, 1 ng of DNA was diluted in 100 μl of water. MtDNA and genomic DNA isolated was subjected to qPCR (standard conditions) using primers against mitochondrial genes cycs, cox3, and 16rs and nuclear encoded gene beta-globin. Primer sequences and run conditions for mitochondrial genes were as previously described (50). The final data was represented as relative amount of mitochondrial gene amplification normalized to nuclear gene amplification.

### Mitochondria membrane potential assay

JC-1 assay (Adipogen Life Sciences, San Diego, CA) was used to assess mitochondrial membrane potential. SSPC were incubated with 0.5 μM of JC-1 reagent in 3% Bovine Serum Albumin (BSA) in PBS for 10 min at 37°C. Cells were washed once with excess PBS and were resuspended in 200 μl of BSA solution. Samples were subjected to FACS analyses where dot plots with axes FITC-A vs. PE-A with positive gates set according to the unstained sample. Using FlowJo software (BD biosciences, San Jose, CA) for analyses, healthy mitochondria were identified as cell populations that were FITC^high^ PE^high^ and unhealthy mitochondria (due to loss of membrane potential) were identified as FITC^high^ PE^low^ population.

### Estimation of ROS levels

Cellular ROS was estimated using the CM-H_2_DCFDA reagent (ThermoFisher). Cells were incubated with 5 μM of the reagent in 3%BSA in PBS at 37°C for 30 min. Cells were washed once with ice-cold PBS and then subjected to FACS analyses. Histogram plots of FITC-Area from 10,000 cells were recorded and mean fluorescence intensity (MFI) were estimated for analyses using FlowJo software.

### RNAscope RNA hybridization

Paraffin embedded tissue sections were used for RNAscope. Probes against mouse *Aldh3a1* and *ckit* were used (ACD bio, Newark, CA). The slides were baked at 70°C for an hour. Slides were deparaffinized with fresh xylene treatment followed by alcohol and finally rehydrated in distilled water. Antigen retrieval step was performed by incubating the slides in 1X RNAscope antigen retrieval buffer at 97°C for 30 min. After cooling down, diluted protease reagent was added on to the sections and incubated at 40°C for 10 min. Sides were washed with distilled water two times for 2 min each. Slides were incubated in the probes (1:50) at 40°C for 16 hours (overnight step). Next day, slides were washed twice in 1X RNAscope wash buffer 2 min each. Three step amplification was carried for 15 to 30 min at 40°C with intermittent washing steps. After the final wash, slides were incubated in washing buffer containing DAPI (1:10,000) followed by mounting cover slips carefully with ProlongGold on glass slides. A total of five to six epithelia images were acquired using a total magnification of 630x using the Leica DMi8 fluorescence inverted microscope. Quantification was done using Qupath software following instructions recommended by the manufacturer.

### ALDH activity assay

Human recombinant ALDH1A1, ALDH1A2, ALDH1A3, ALDH1B1, ALDH2, ALDH3A1, ALDH3A2, and ALDH4A1 were expressed and purified using nickel column chromatography as previously described (51). The assay buffer was 100 mM sodium phosphate (pH 8.0) with 1 mM MgCl_2_. Reaction was conducted in the white opaque 96-well assay plates (Corning Costar, flat-bottom, and nontreated) with a total reaction volume of 100 μl for each well. Each reaction mixture consisted of 100 nM ALDH enzyme, 0.1% DMSO, 1 mM β-mercaptoethanol, 1 mM NAD+, 1 mM acetaldehyde (lastly added), and CB29 of indicated concentration in assay buffer. Enzymatic activity was measured based on NADH-mediated fluorescence (Ex340/Em460 nm). The activity was recorded for 10 min on a SpectraMax M2e microplate reader operated by the SoftMax Pro software. ALDH enzymatic activity caused by CB29 was normalized to the DMSO control. The single-dose inhibition data and dose–response inhibition curves were processed with the Prism software.

### LC/MS analyses of metabolites

Freshly isolated cells from murine WT and *Aldh3a1^–/–^* SMGs were used from LC/MS analyses. Please refer to Supplemental methods for details.

### siRNA transfection

MSGc (120,000/well) were plated in a 6-well format. A total of 24 hours later, cells were transfected with 30 pmoles of siAldh3a1 (Ambion, AM16708) and scramble siRNA using Lipofectamine 3000 following Manufacturers recommended protocol. A total of 24 hours later, cells were trypsinized, counted, and used for downstream analyses.

### Irradiation of salivary glands and salivary collection

A total radiation dose of 30 Gy was delivered in five fractions (6 Gy/fraction/day) to the SMG with the rest of the body lead shielded. Mice were treated with 10% Alda-341 mixed in daily chow or no treatment. Stimulated saliva was measured as previously described (6). Mice were anesthetized with a ketamine (80 mg/kg) and xylazine (16 mg/kg) mixture delivered by intraperitoneal injection and subcutaneously injected with 2 mg/kg pilocarpine. Saliva was collected for 15 min. Saliva volume was calculated by assuming the density as 1, was normalized to the mouse body weight by dividing the total collected saliva volume by the mass of the mouse (kg).

### Study approval

Human salivary glands were procured from HNC patients in accordance with the guidelines approved by the Stanford University's Institutional Review Board. Written informed consent was received from participants prior to inclusion in our study. The human tissue was rinsed twice with diluted betadine solution and washed with excess sterile phosphate buffered saline (PBS) three times before dissociation.

### Statistics

All data are represented as averages with standard deviation (SD). Statistical ANOVA and Student's t tests were used to compare the data. All tests performed were two-sided with an alpha level of 0.05. *P* ≤ 0.05 was considered to be significant. All data was analyzed using GraphPad Prism 8.4.3 (GraphPad Software Inc, La Jolla, CA).

## Supplementary Material

pgac056_Supplemental_File

## Data Availability

Data generated in this study is available on public open access repository Dyrad (https://doi.org/10.5061/dryad.9w0vt4bgg).
