# Correction to: Aldehyde dehydrogenase 3A1 deficiency leads to mitochondrial dysfunction and impacts salivary gland stem cell phenotype

**DOI:** 10.1093/pnasnexus/pgad460

**Published:** 2024-01-30

This is a correction to: Vignesh Viswanathan, Hongbin Cao, Julie Saiki, Dadi Jiang, Aaron Mattingly, Dhanya Nambiar, Joshua Bloomstein, Yang Li, Sizun Jiang, Manish Chamoli, Davud Sirjani, Michael Kaplan, F Christopher Holsinger, Rachel Liang, Rie Von Eyben, Haowen Jiang, Li Guan, Edward Lagory, Zhiping Feng, Garry Nolan, Jiangbin Ye, Nicholas Denko, Sarah Knox, Daria-Mochly Rosen, Quynh-Thu Le, Aldehyde dehydrogenase 3A1 deficiency leads to mitochondrial dysfunction and impacts salivary gland stem cell phenotype, *PNAS Nexus*, Volume 1, Issue 2, May 2022, pgac056, https://doi.org/10.1093/pnasnexus/pgac056

During a retroactive audit conducted by *PNAS Nexus*, it was discovered that this paper was missing a statement acknowledging compliance with the *PNAS Nexus* Human and Animal Participants and Clinical Trials policy:

Our full study protocol involving human participants was approved by the Stanford IRB panel 3 under the protocol number 11977.

This error has been corrected in the original article.

