# Supplementary material for: Aldehyde dehydrogenase 3A1 deficiency leads to mitochondrial dysfunction and impacts salivary gland stem cell phenotype
Source: PNAS Nexus. 2022 Jun 9;1(2):pgac056. doi: 10.1093/pnasnexus/pgac056 (PMC9186046; doi:10.1093/pnasnexus/pgac056)

**Supplementary methods:**

**Drugs used:**

Alda-341 (d-limonene) and *N*-Acetyl-L-cysteine (NAC) was purchased from Sigma-Aldrich (St. Louis, MO). Carbonyl cyanide 4-(trifluoromethoxy)phenylhydrazone (FCCP), 4-hydroxynonenal (4-HNE), ALDH3A1 inhibitor CB29 were purchased from MilliporeSigma (Burlington, MA) and reconstituted in sterile DMSO (FisherScientific, Hampton, NH). MitoTEMPO was purchased from MilliporeSigma and dissolved in molecular grade sterile water. Hydrazine derivative Hydralazine hydrochloride was purchased from TCI America (Portland, OR) and dissolved in molecular grade water (Thermo Fisher Scientific, Waltham, MA).

**Salivary gland isolation and culture from mouse and human SMGs**:

With sterile clean forceps and surgical scissors, a small cut (0.5 inch) was made vertically below the jaw line to expose the submandibular glands. Connective tissue was removed from the glands with the help of sharp forceps. The gland was minced into small pieces by using a clean surgical blade. The minced gland tissue (mouse or human) was transferred into a 15-ml tube containing 3-6 ml of dissociation media (DMEM/F12 with collagenase (0.025%) and hyaluronidase (0.04%), 6.25 mM CaCl_2_, and fungizone. Tissue dissociation was allowed for an hour at 37 C on a shaker. Equal amount of Dispase I enzyme (CORNING, Corning, NY) was added to the dissociation media and incubated for another hour. The dissociated cells were passed through a 100-micron sterile filter and were spun down at 1200 rpm for 5 minutes. After removing the supernatant, RBCs were lysed by incubating the cell suspension in ACK RBC lysis buffer (LONZA, Basel, Switzerland) for 2 minutes at room temperature. The buffer was neutralized by adding media containing FBS and cells were spun down again. To the cell pellet, 0.25% trypsin-EDTA solution was added and incubated for 1 minute at 37 C to facilitate single cell dissociation. After neutralizing with stem cell media, the cell suspension was passed through a sterile 70-micron filter. For human sample, The cells were spun down and re-suspended in required amount of salisphere media (DMEM/F12 + GlutaMax media containing 10% FBS, 1x antibiotic-antimycotic, 1% N2 supplement, 20 ng/mL epidermal growth factor-2, 20 ng/mL fibroblast growth factor-2, 10 μg/mL insulin, 1 μM dexamethasone, 10 μM Y-27632). Cell suspension after gland dissociation was mixed with twice the volume of Growth factor reduced matrigel (CORNING) and 75 μl of the mixture was added as a drop to a 12-well plate. The mixture was incubated at 37 C for 20 minutes to allow it to solidify followed by addition of 1 ml of salisphere media. Dissociated murine salivary gland cells were blocked in 3% BSA in PBS and stained for live cells (Zombie NIR dye, 1:1000) for 10 minutes on ice. Cells were washed once with cold blocking buffer and resuspended in an antibody cocktail containing anti-EpCAM-FITC, anti-CD24-APC, anti-CD31-PE, and anti-CD45-PE (1:200) in blocking buffer and incubated for 10 min on ice. Cells were washed with excess blocking buffer, resuspended, and subjected to FACS analyses and sorting using a BD FACS ARIAII. Unstained cell sample were used to gate the positive cells with the FACS DIVA software (BD, San Jose, CA). Cells that stained negative for zombie dye followed by negative for CD45 (immune marker), CD31 (endothelial cells) expression were selected and EpCAM^high^ /CD24^high^ cells were sorted. Matrigel was plated on bottom of a 48 well plate (CORNING) and allowed to solidify at room temperature. Cell suspension containing sorted cells in salisphere media were plated on top of the matrigel (10,000 cells per well). The spheres were passaged on day 7. For passaging, matrigel containing spheres were spun down and then incubated in Dispase I solution for 45 minutes. Spheres were separated by spinning the solution down and aspirating the supernatant carefully. The spheres were dissociated by incubating them in 0.25% trypsin for 10 min followed by shearing by a 27G needle syringe. The single cells were spun down and re-suspended in sphere media ready to be plated. For quantification, brightfield images of the spheres were taking by BZ-X710 KEYENCE microscope using the z-stack function. Images were stitched together into a single image and counted by ImageJ software (NIH, Bethesda, MD).

**RNA Sequencing:**

RNA samples were extracted using Qiagen miRNeasy kit (QIAGEN, Hilden, Germany). The cDNA was generated from extracted RNA using the Smarter Ultra Low Input RNA kit (TakaraBio, Shiga, Japan). The amplified cDNA was purified and then sheared into an avg 300 bp length using Covaris S2. Libraries were generated from the sheared cDNA using Clontech Low Input Library Prep kit. Sequencing data was generated using Illumina Hi Seq 4000 system. The processed reads were imported into BRB ArrayTools, an integrated package for the visualization and statistical analysis of gene expression data developed by Dr. Richard Simon and BRB-Array Tools Development Team (Biometric Research Program, National Cancer Institute).The log_2_ fold change of gene expression between the two samples were calculated using EdgeR package. MetaCore (GeneGo) was used for Gene ontology analyses of the differentially expressed genes between the two groups. For the groups WT SSPC vs (WT+Alda-341), gene counts were uploaded on Biojupies (47) to generate heatmap of differentially expressed genes and GO term classification.

**Embryo gland dissection and branching morphogenesis assay:**

The glands were carefully fixed in 4%PFA for 10 min followed by a wash with PBS. They were carefully embedded in 200 μl of pre-warm of Histogel (Thermo-Fisher). Once the gel solidified, it was moved to 70% ethanol and process for paraffin embedding. For branching morphogenesis assay, isolated E13.5 epithelia and mesenchyme were separated using Dispase treatment and mechanical dissection and cultured in a drop of laminin on a nucleopore filter over serum-free DMEM/F12 containing transferrin and ascorbic acid (complete media) as described for the E13.5 SMG . Epithelia were cultured with 400 ng/ml FGF10 (R&D Systems, Minneapolis, MN) and 0.5 μl/ml heparin sulfate (Sigma-Aldrich) in the presence or absence of Alda-341 or vehicle control (PEG-400) and were subjected to RNA isolation or fixed for immunostaining after 24-48 h.

**Immunostaining:**

Sections from paraffin embedded salivary gland tissue or spheres were used for Immunostaining. Slides were de-paraffinized by 2 rounds of incubation in fresh xylene for 10 min each. The slides were sequentially rehydrated by washing them in increasing concentration of water in alcohol for 3 min each. Antigen retrieval was performed by boiling the slides in 1x Antigen retrieval solution (Vector labs, Burlingame, CA) using rice cooker for 12 minutes. After cooling down at room temperature slides were washed twice with PBS. For intracellular staining, slides were incubated in 0.1% Tween-20 in PBS (PBS-T) for 10 min and washed twice again with PBS. For immunohistochemistry (IHC), an addition step of treatment of slides with 3% hydrogen peroxide in methanol for 10 min was added followed by PBS wash. Slides were incubated blocking buffer for 1 hour at RT followed by overnight incubation at 4 C in the primary antibodies. List of antibodies and dilutions are provided in **Table S1.** Next day, slides were washed in PBS three times and then incubated in secondary antibody for 1 hr. Following secondary antibody incubation, slides were washed three times in PBS and Gold-anti FADE mounting media containing DAPI was added as a counterstain for immunofluorescence (IF). For IHC, slides for incubated in activated DAB for 30-60 seconds and followed by multiple washes with distilled water. Slides were then de-hydrated by treating with decreasing amount of water in alcohol and then to xylene for 30 seconds each. Images were taken from ten random field of view using the Leica Dmi8 fluorescence microscope. These images were used for quantifications using ImageJ software (NIH, Bethesda, MD). For fluorescence staining, the intensity was represented as mean fluorescence intensity (MFI) and for immunohistochemistry, it was represented as mean arbitrary units (A.U.).

**Western Blotting:**

Protein was isolated from cells using 1X RIPA lysis buffer (EMD Millipore, Burlington, MA) containing 1X protease inhibitor cocktail (Thermo-Fisher, Waltham, MA). Protein concentrations were estimated using Pierce BCA assay kit (Thermo-Fisher). Thirty micrograms of protein were loaded in Mini-Protean precast gels (Bio-Rad, Hercules, CA). Gel electrophoresis was run at a steady voltage of 75 V at 4^0^C. After the gel run was completed, resolved proteins were transferred on a nitrocellulose membrane using the Turbo Transblot kit (Bio-Rad) following the manufacturer’s protocol. The blots were incubated in 3% BSA in TBS-T for 1 hours for blocking followed by overnight incubation in primary antibody. Next day, the blots were washed thrice in 0.1% Tween-20 in Tris Buffed Saline (TBS-T) for 5 minutes each. HRP conjugated secondary antibody was prepared in TBS-T (1:1000) added to the blots for incubation for 1 hour. Excess unbound secondary antibody was removed by three washes of TBS-T. Blots were developed by using Super Signal West Pico plus Chemiluminescent substrate (Thermo-Fisher).

**PI/Annexin V assay:**

Cells were washed with cold PBS and incubated with anti-Annexin V-FITC diluted solution (Bio-legend, San Diego, CA) for 10 minutes at 37^0^C in the dark followed by PI solutions for 5 min. Cells were washed again with cold PBS and subjected to FACS analyses using BD LSR FortessaX-20 flow cytometer. Using FACS DIVA software, positive quadrant gates in the plots were determined using unstained cells as a control sample. Cell populations were identified as early apoptosis (Q1:Annexin V ^+ve^/ PI^-ve^), late apoptosis (Q2:Annexin V^+ve^/ PI^+ve^) and total apoptosis as the sum of all three quadrants (Q1,Q2,Q3). Changes in apoptosis were analyzed after 24-48 hours of treatment with drugs (FCCP, CB29, mito-TEMPO, 4-HNE, Hydralazine) or vehicle control. FlowJo (Ashland, OR) was used for analyses and data was represented as average percent apoptosis in the treatment normalized to control.

**EM imaging:**

Samples were fixed in Karnovsky’s fixative: 2% Glutaraldehyde (EMS, Sumter, SC) and 4% paraformaldehyde (EMS) in 0.1M Sodium Cacodylate (EMS) pH 7.4 for 1 hr.  The fix was replaced with cold/aqueous 1% Osmium tetroxide (EMS Cat# 19100) and were then allowed to warm to Room Temperature (RT) for 2 hrs rotating in a hood, washed 3X with ultrafiltered water, then stained in 1% Uranyl Acetate at RT 2hrs while rotating.  Samples were then dehydrated in a series of ethanol washes for 30 minutes each @ RT beginning at 50%, 70% EtOH then moved to 4^o^C overnight.  They were placed in cold 95% EtOH and allowed to warm to RT, changed to 100% 2X, then Propylene Oxide (PO) for 15 min.  Samples are infiltrated with EMbed-812 resin (EMS) mixed 1:2, 1:1, and 2:1 with PO for 2 hrs each with leaving samples in 2:1 resin to PO overnight rotating at RT in the hood.  The samples are then placed into EMbed-812 for 2 to 4 hours then placed into molds w/labels and fresh resin, oriented, and placed into 65^o^ C oven overnight. Sections were taken around 80nm, picked up on formvar/Carbon coated slot Cu grids, stained for 40seconds in 3.5% Uranyl Acetate in 50% Acetone followed by staining in Sato’s Lead Citrate for 2 minutes.  Observed in the JEOL JEM-1400 120kV.  Images were taken using a Gatan Orius 832 4k X 2.6k digital camera with 9 um pixel.

**LC/MS analyses:**

Cells were washed with warm media and [U-^13^C]glucose RPMI medium (10% dialyzed FBS) lacking glucose, serine, and glycine (TEKnova, Hollister, CA) and reconstituted with [U^13^C]glucose (2 g/liter), serine (0.03 g/liter), and glycine (0.01 g/liter) was added to each well and incubated for 6 hours. Cells were washed twice with ice-cold PBS prior to extraction with 80:20 acetonitrile: water over ice for 15min, sonicated for 30s Biorupter 300 (Diagenode) sonicator, then spun down at 1.5 x 10^4^ RPM for 10 min. 200 µL of supernatant was taken out of for the LC-MS/MS analysis immediately. Quantitative LC-ESI-MS/MS analysis of ^13^C-glucose-labeled cell extracts was performed using an Agilent 1290 UHPLC system equipped with an Agilent 6545 Q-TOF mass spectrometer (Santa Clara, CA, US). A hydrophilic interaction chromatography method (HILIC) with an BEH amide column (100 x 2.1 mm i.d., 1.7 μm; Waters) was used for compound separation at 35 °C with a flow rate of 0.3ml/min. The mobile phase A consisted of 25 mM ammonium acetate and 25mM ammonium hydroxide in water and mobile phase B was acetonitrile. The gradient elution was 0 – 1.5 min, 80%B; 1.5 – 7 min, 80%B → 40%B; 7 – 8.5 min, 40%B; 8.5 – 8.7 min, 40% → 80%B; 8.7 – 10 min, 80%B. The overall runtime was 10 min and the injection volume was 6 μL. Agilent Q-TOF was operated in negative mode and the relevant parameters were as listed: ion spray voltage, 3500 V; nozzle voltage, 1000 V; fragmentor voltage, 125 V; drying gas flow, 11 L/min; capillary temperature, 300 °C, drying gas temperature, 320 °C; and nebulizer pressure, 40 psi. A full scan range was set at 50 to 1200 (m/z). The reference masses were 119.0363 and 980.0164. The acquisition rate was 2 spectra/s. Data processing was performed with Agilent Profinder B.08.00 (Agilent technologies). The mass tolerance was set to +/- 15 ppm and RT tolerance was +/- 0.2 min. Natural isotope abundance was corrected using Agilent Profinder software (Agilent Technologies). For normalization of ion counts, cell pellets were vacuum-dried, then protein concentration was determined by Pierce^TM^ BCA protein assay kit (Thermo-Fisher) according to manufacturer’s instructions.

**Supplementary Figure legends:**

**Supplementary Figure 1: A)** Mice were treated with 30 Gy + Alda-341 continuously for 18 weeks and observed for another 4 weeks without any drug treatment (total of 22 weeks from radiation) or 30 Gy + no drug, N=7-8 per group. Figure shows salivary index (saliva volumes/gm of body weight) of the mice receiving radiation alone at week 22 (black), and the Alda-341-treated mice at week 18 (last day of Alda-341 treatment, red), and at week 22 (4 weeks after stopping drug treatment, grey). One-way ANOVA with multiple comparison was used to calculate the p value (** represents p value < 0.01). **B)** Tissue morphology of major murine salivary glands in WT and *Aldh3a1^-/-^* mice represented by hematoxylin and eosin staining imaged at 100x total magnification (n=3/group). Scale bar: 100 μM.

**Supplementary Figure 2:** SEURAT analyses of embryonic, postnatal and adult murine SMG epithelium (48). **A)** UMAPs of Aldh3a1 expression across individual cluster of cells in embryonic (E12, E14 and E16), postnatal (P1 and P30) and adult murine SMGs. **B)** Violin plot of Aldh3a1 expression across identified cell clusters in postnatal and adult murine SMGs. Red boxes indicate Aldh3a1 expression identified in ductal cell clusters.

**Supplementary Figure 3: A)** Representative immunohistochemistry images of SMG derived for 3 different human patients stained for ALDH3A1 (200x total magnification). **B)** Representative images for vehicle control (left) and 200 μM Alda-341 (right) after 24 h in culture and immunoassayed with c-KIT (green), ECAD (red), and DAPI (blue), and imaged with a confocal microscope. Ten μM confocal section of all three markers (top) and averaged sections of c-KIT only (bottom)**. C)** Schematic of SSPC isolation and culture from murine SMG. **D)** Heatmap showing differences in the gene expression profiles between WT and *Aldh3a1^-/-^* sorted salivary stem cells (n=3/group). **E)** Gene Ontology MetaCORE analysis of the top 175 differentially regulated genes (FDR < 0.1) showing enriched differential pathways between WT and *Aldh3a1^-/-^* SSPC.

**Supplementary Figure 4: A)** Average log fold change in mitochondrial gene in WT SSPC as compared to *Aldh3a1^-/-^* .**B)** Quantitative PCR analyses of mitochondrial genes in murine SSPC derived from WT and *Aldh3a1^-/-^* SMGs (n=3 mice per group). **C)** Mitochondrial DNA copy represented by DNA amount of mitochondrial encoded genes 16rs, cox3 and cycs normalized to DNA amount of nuclear encoded gene beta-globin as assessed by PCR (n=3 mice/group). **D)** Representative immunostaining images of Tom20 in WT and *Aldh3a1^-/-^* salispheres at 200x and 630x total magnification (scale bar: 250 μM for 200x image, 75 μM for 630x). **E)** Representative TEM images of ductal and acinar cells in WT SMGs (n=3). The dotted white lines represent the ductal cells (top left panel) and acinar cells (top right panel). Magnified images (6000x) of ductal cell (lower left) and acinar cell (lower right) showing much less mitochondria abundance (white arrow) in acinar cells as compared to ductal cells. Two-way ANOVA with multiple comparison was used to calculate the p value for panel **B**. Multiple unpaired t-test was used to calculate p value for panel **C**  (* represents p value < 0.05, ** < 0.01).

**Supplementary Figure 5 A)** Heatmap of differentially expressed genes in WT SSPC treated with Alda-341and vehicle control. **B)** GO term classification of top differentially expressed genes between the two groups identified “mitochondrion” (GO:0005739) to be significantly upregulated in drug treated group as compared to vehicle control.

**Supplementary Figure 6: A)** Dose response curve of CB29 on ALDH3A1 activity **B)** Specificity of CB29 (20 uM) in inhibition of ALDH3A1/2 activity demonstrated by enzyme activity assays of various ALDH isoforms (n=2, 4 technical replicates). **C)** FACS plots showing Annexin-V/PI staining of mSGc 24 hours post treatment with CB29 and vehicle control, quantified and displayed as graph in right panel (n=3, in triplicates). **D)** Average number of spheres counted at day 7 post treatment with 20 uM CB29 and vehicle control (n=2, 3 technical replicates). **E)** Western blot probed for Aldh3a1 and actin in protein isolated from mSGc treated with siAldh3a1 and control. F) FACS plots showing Annexin-V/PI staining of mSGc 24 hours post transfection with siAldh3a1 and control, quantified and shown in the right panel (n=2, in triplicates). Error bars represent SD. Student’s t-test was used to calculate the p value for panel **C, D** and **F.** One-way ANOVA with multiple comparison was used to determine p value for panel **B**. (** represents p value < 0.01, *** < 0.001)

**Supplementary Figure 7:** **A)** Representative bright field images of WT and *Aldh3a1^-/-^* spheres at day 7 treated with NAC and vehicle control. Image J was used to count the number of spheres and represented in the right panel. **B)** Carbon labelling experiments demonstrate differences in acetyl-coA influx in WT and *Aldh3a1^-/-^* murine SSPC (n=3/group). Error bars represent SD. Two-way ANOVA with multiple comparison was used to calculate p value for **A** right panel. Students t test was used to estimate the p value for **B**. (* represents p value < 0.05, ****< 0.0001)

**Supplementary Figure 8: A)** Illustration representing potential role of ALDH3A1 in regulating mitochondrial function and survival of murine SSPC. ROS a derivative of mitochondrial activity, when accumulated can mediate lipid peroxidation (LPO) to make 4-HNE. 4-HNE, one of the major substrates of ALDH3A1 is broken down to fatty acids. ALDH3A1 converts GSSG to GSH which can act as an antioxidant to reduce oxidative stress, In ALDH3A1 deficient cells, GSH depletion by itself can contribute to poor survival. Overall ALDH3A1 deficiency or loss of function can lead to reduction of GSH reserve, accumulation of ROS and its by-products 4-HNE that can impair mitochondrial function to reduce overall survival of murine SSPC. **B)** Representative immunofluorescence image of gammaH2AX staining in WT and *Aldh3a1^-/-^* SMG (n=3/group). **C)** Q-PCR results showing normalized fold change in expression of two DNA damage response genes *p21* and *Bax* in WT and *Aldh3a1^-/-^*  derived SSPC (n=3/group). Error bars represent SD. Students t test was used to estimate the p value for **C**. (* represents p value < 0.05)

**Table S1:** List of antibodies used in the study with their respective source, dilution, and application.


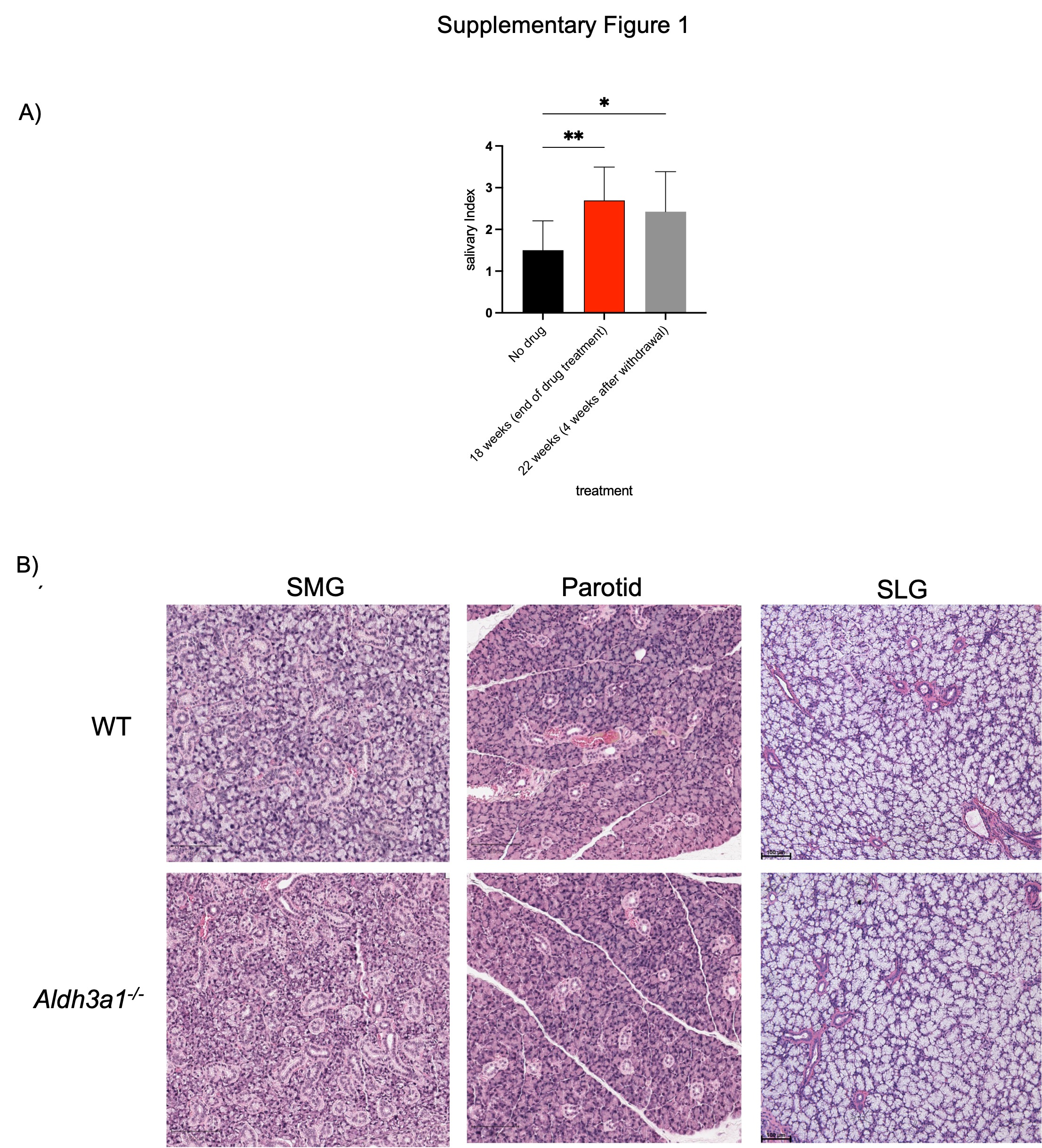


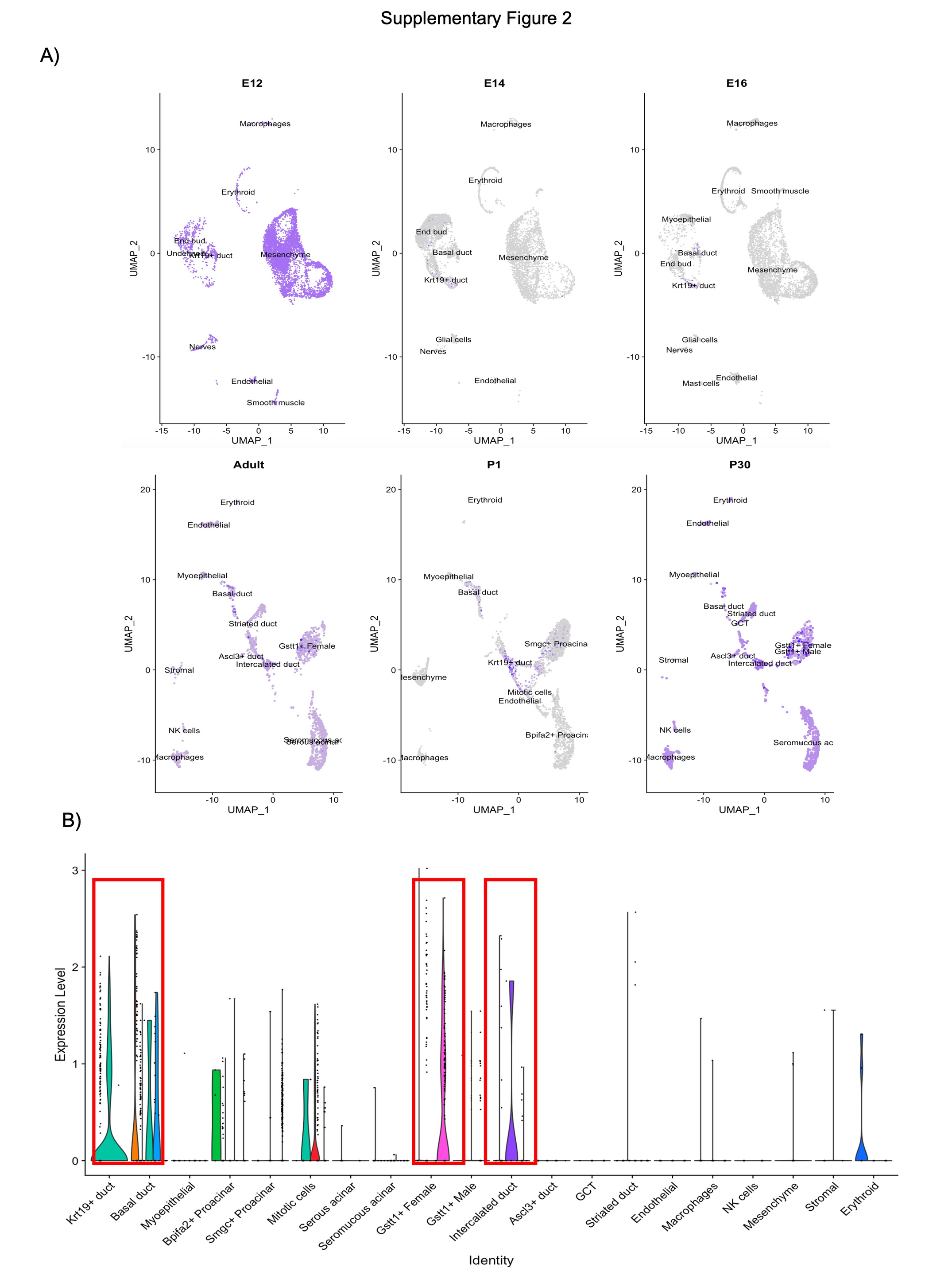


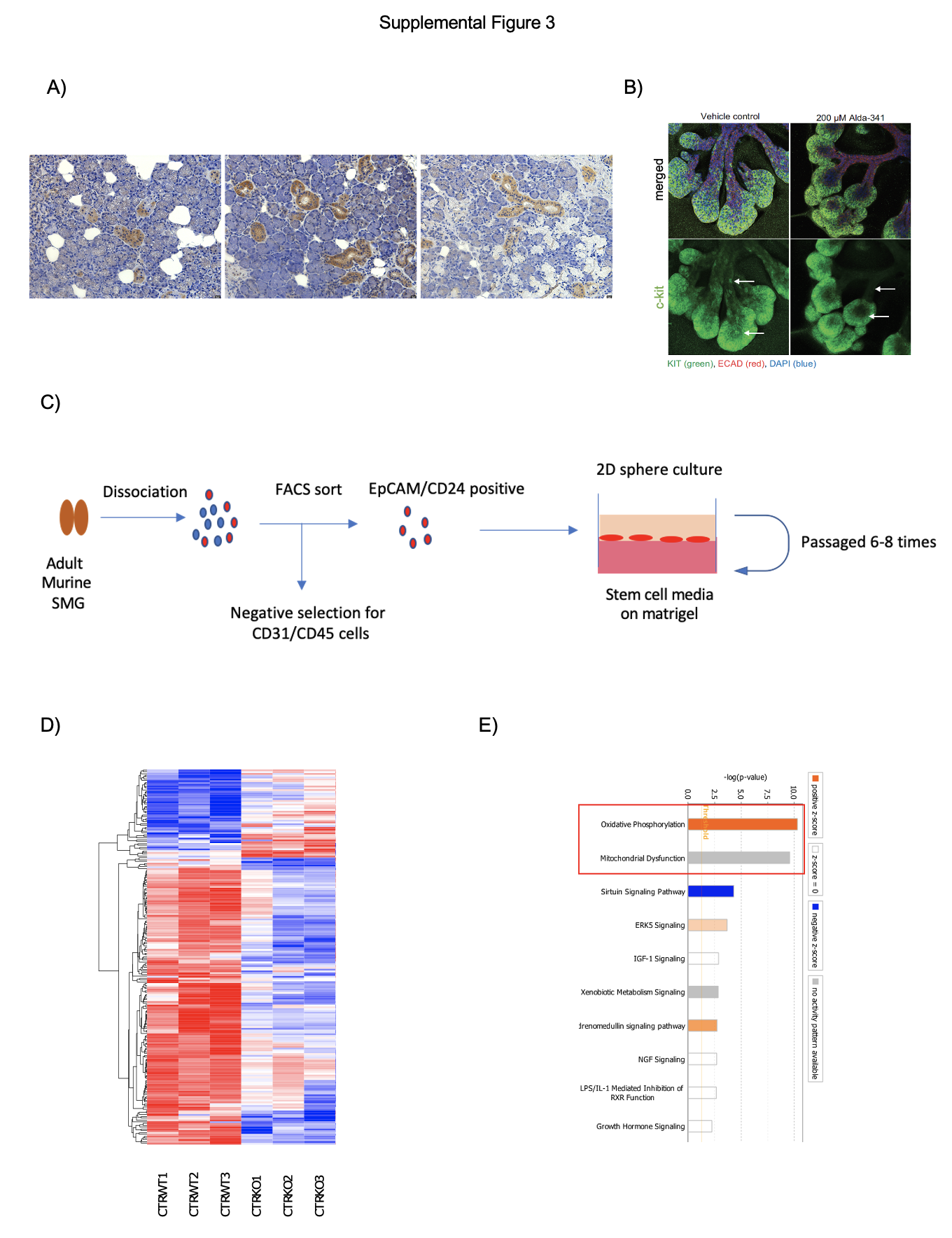


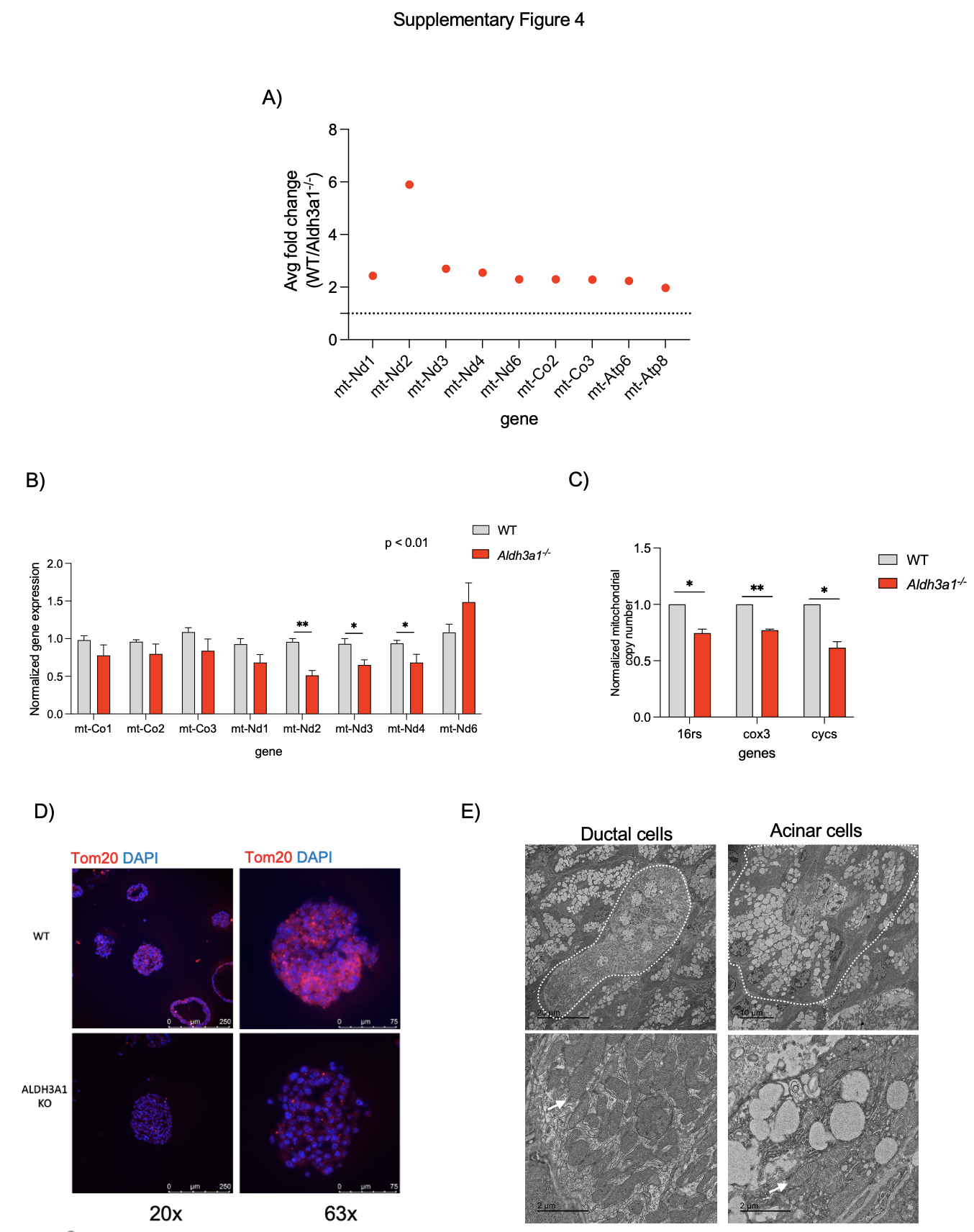


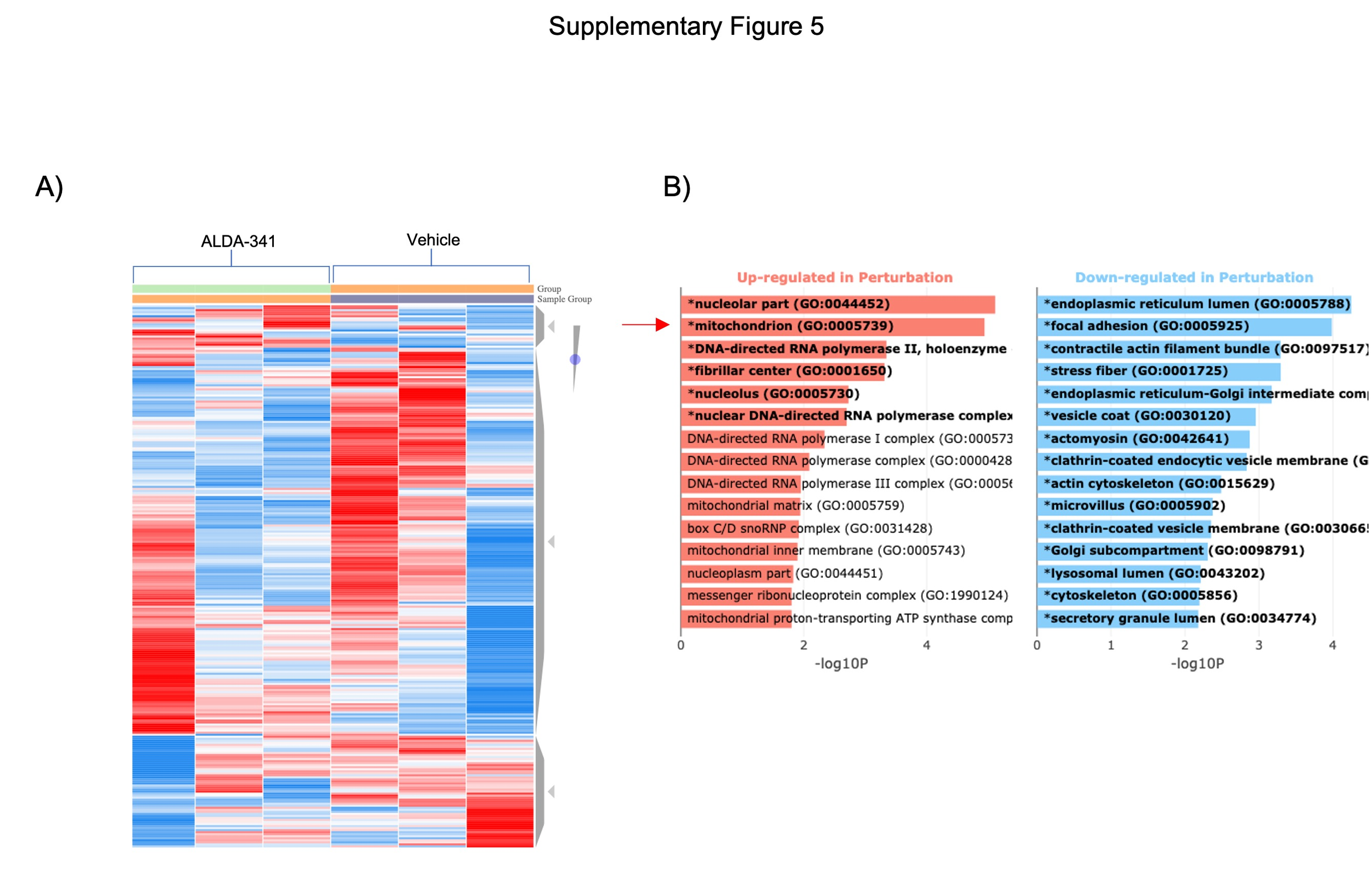


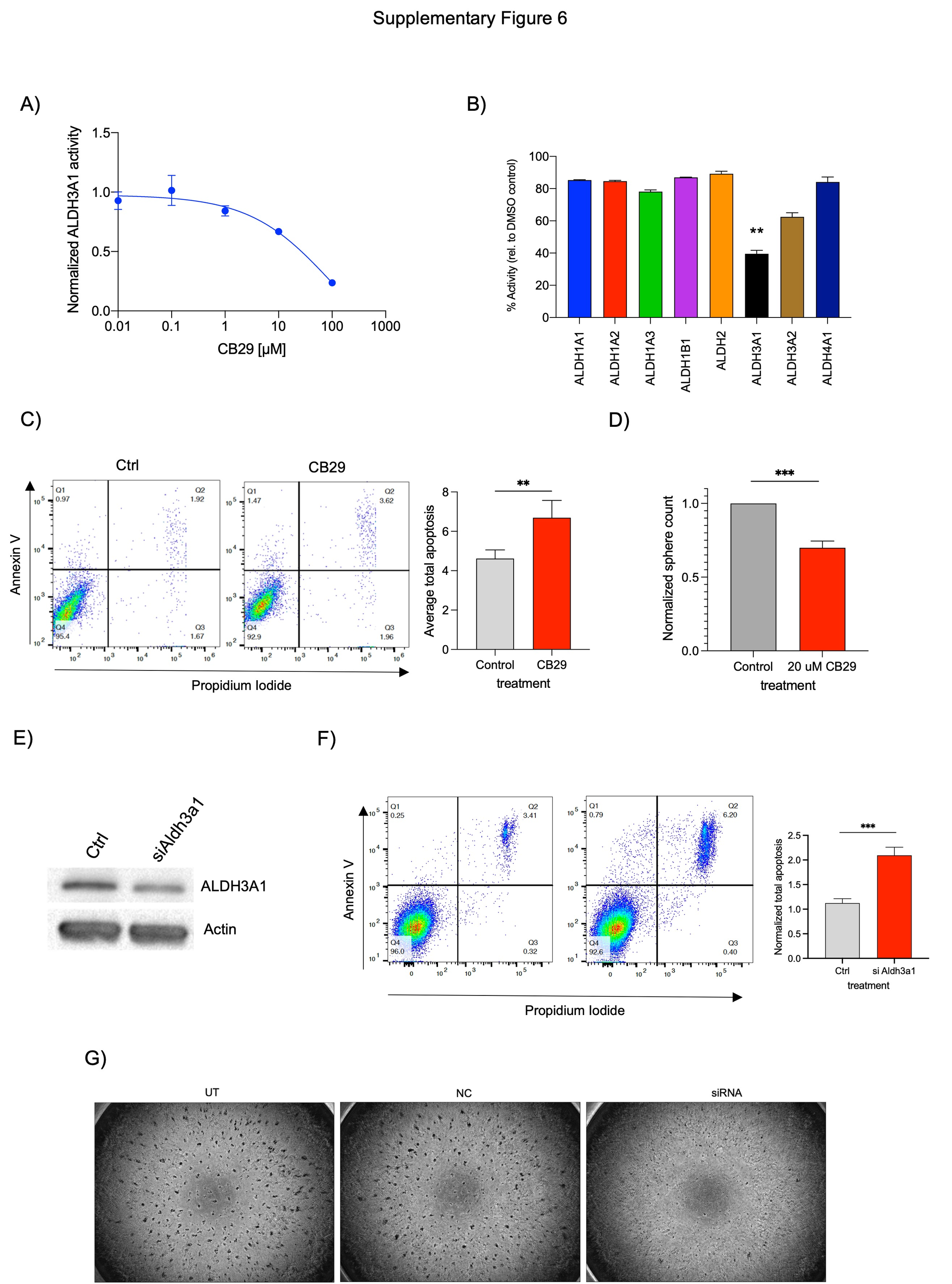


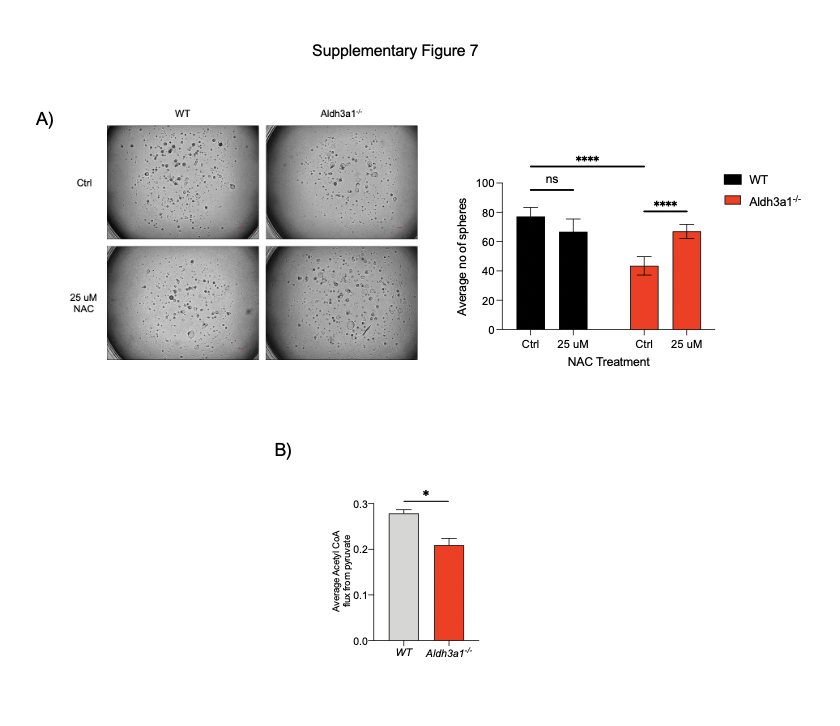


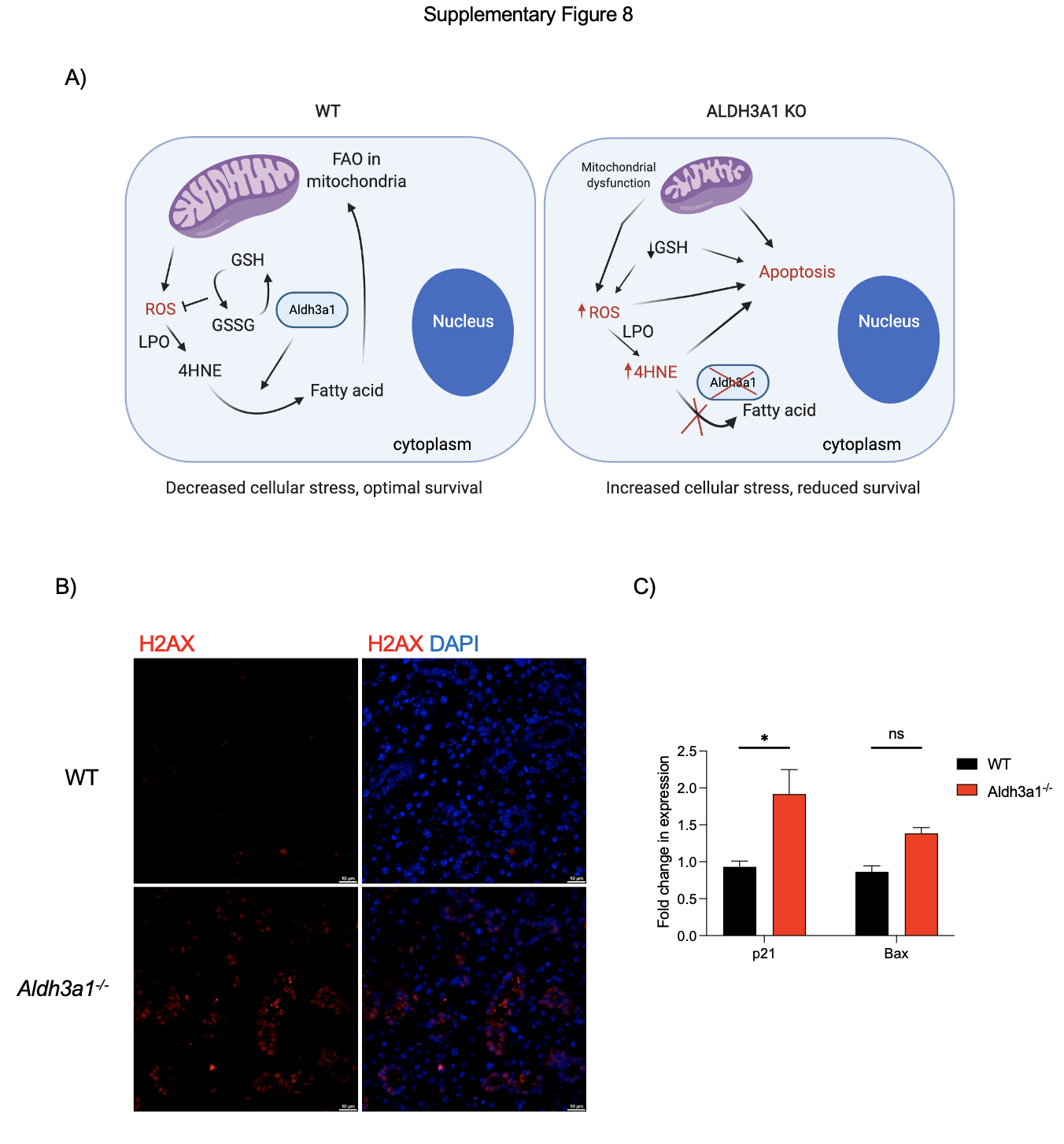


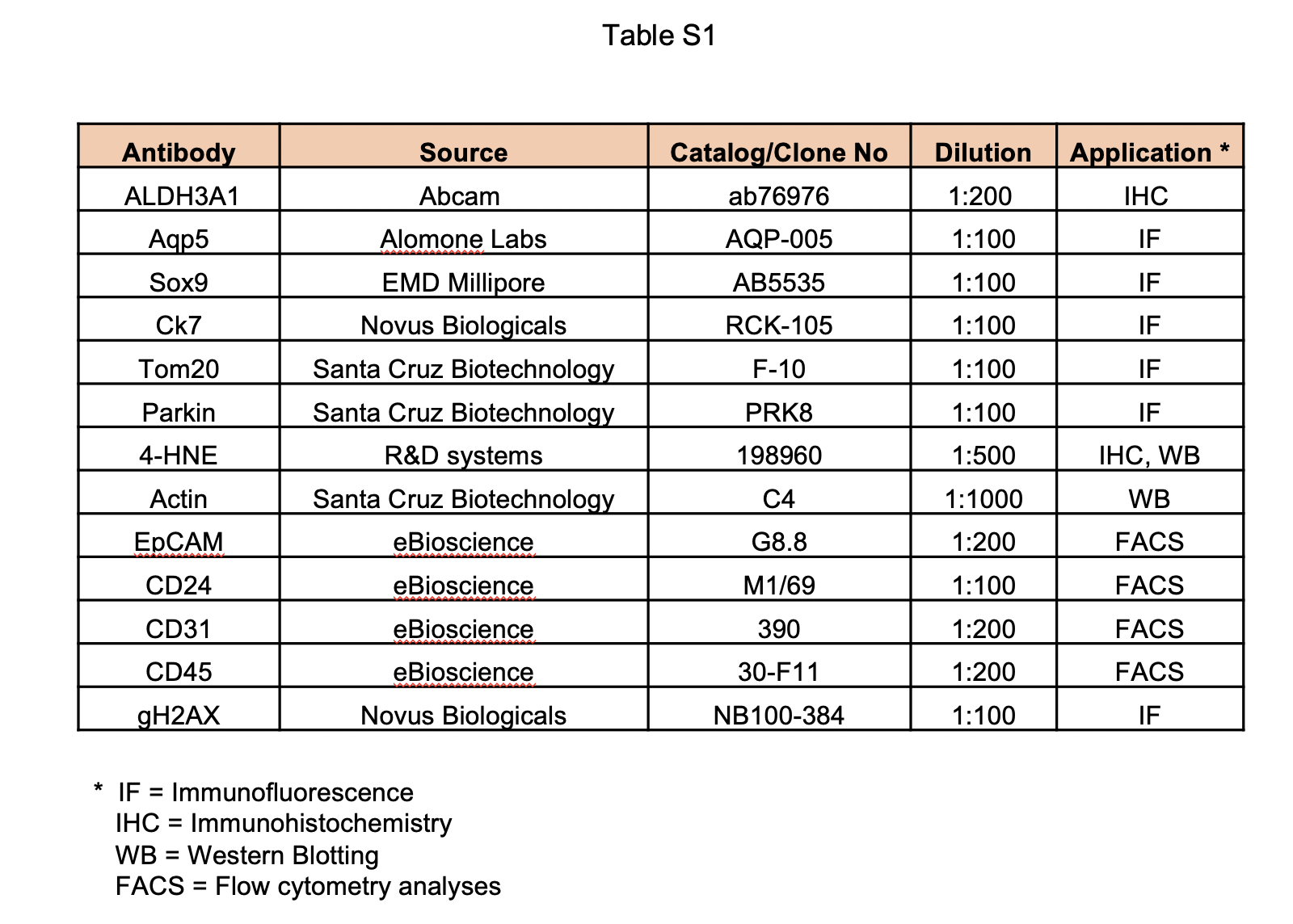

Supplement: pgac056_Supplemental_File [file pgac056_supplemental_file.docx]
